# Biomarker potential of the LEF1/TCF family members in breast cancer: Bioinformatic investigation on expression and clinical significance

**DOI:** 10.1590/1678-4685-GMB-2022-0346

**Published:** 2023-12-15

**Authors:** Beatriz Miotto Lima, Alexandre Luiz Korte de Azevedo, Igor Samesima Giner, Talita Helen Bombardelli Gomig, Enilze Maria de Souza Fonseca Ribeiro, Iglenir João Cavalli

**Affiliations:** 1 Universidade Federal do Paraná, Departamento de Genética, Curitiba, Paraná, Brasil.

**Keywords:** Breast cancer, LEF1/TCF family, Biomarkers, Bioinformatics

## Abstract

The LEF1/TCF transcription factor family is related to the development of diverse tissue types, including the mammary tissue, and dysregulation of its expression and function has been described to favor breast tumorigenesis. However, the clinical and biological relevance of this gene family in breast cancer is still poorly understood. Here, we used bioinformatics approaches aiming to reduce this gap. We investigated its expression patterns in molecular and immune breast cancer subtypes; its correlation with immune cell infiltration, and its prognostic values in predicting outcomes. Also, through regulons construction, we determined the genes whose expression is influenced by these transcription factors, and the pathways in which they are involved. We found that *LEF1* and *TCF3* are over-expressed in breast tumors regarding non-tumor samples, while *TCF4* and *TCF7* are down-expressed, with the gene’s methylation status being associated with its expression dysregulation. All four transcription factors presented significance at the diagnostic and prognostic levels. *LEF1*, *TCF4,* and *TCF7* presented a significant correlation with immune cell infiltration, being associated with the immune subtypes of less favorable outcomes. Altogether, this research contributes to a more accurate understanding of the expression and clinical and biomarker significance of the LEF1/TCF transcription factors in breast cancer.

## Introduction

The T-cell factors/lymphoid enhancer-binding factors *LEF1* (*TCF1α*), *TCF3* (*TCF7L1*), *TCF4* (*TCF7L2*), and *TCF7* (*TCF1*) represent the LEF1/TCF family, a group of nuclear DNA-binding transcription factors. These proteins regulate the expression of a large sum of targets through their multiple binding sites and splicing variants (Arce et al. 2006), influencing several biologic processes, including embryonic patterning, tissue homeostasis, and cell fate determination (Hrckulak et al., 2016). As effectors of the canonical Wnt signaling pathway, the LEF1/TCF members participate in the genetic circuits involved in the development of the mammary gland and breast tissue (Boras-Granic et al., 2006; Abreu de Oliveira et al., 2022). Alterations in its expression and function can lead to the dysregulation of several biological processes and, consequently, to micro and macro alterations in breast biology, including the development of neoplasia (Boras-Granic and Hamel 2013; Yu et al., 2016). 

It has been appointed that the LEF1/TCF transcription factors can act in tumorigenesis via regulation of metastasis and invasion (Li et al., 2014; Chen et al., 2018; Blazquez et al., 2020); cell cycle (Cordray and Satterwhite, 2005); proliferation (Hao et al., 2019); apoptosis and chemosensitivity (Xie et al., 2012), and regulation of immune system elements (Xing et al., 2019). Moreover, the expression of LEF1/TCF transcription factors can be associated with prognosis and treatment response in various cancer types, such as colorectal and liver cancer (Lin et al., 2011; Li *et al.*, 2014; Anwar et al., 2020), oral squamous cell carcinomas (Su et al., 2014), acute lymphoblastic leukemia (Fischer et al., 2015), prostate cancer (Chen *et al.*, 2018), and lung cancer (Zhu et al., 2015). In breast cancer, the LEF1/TCF family members also have a distinctive role in tumorigenesis. LEF1 and TCF4 dysregulated expression, for example, was associated to cell proliferation and invasion through Wnt pathway alterations (Nguyen et al., 2005; Ravindranath et al., 2011; Sergio et al., 2020); while TCF3 was associated with tumor growth, proliferation, and stem cell self-renewal (Slyper et al., 2012; Jia et al., 2020), and TCF7 to brain-seeking breast metastasis (Park et al., 2015). However, its clinicopathological and predictive values, expression pattern, and biomarker potential are still largely unknown.

In 2020, breast cancer assumed the rank of the most diagnosed cancer worldwide, surpassing lung cancer; among women, breast cancer is also the leading cause of cancer death (Sung et al., 2021). Breast cancer can be subdivided according to molecular subtypes (Sørlie et al., 2003) and immunohistochemical subtypes (Goldhirsch et al., 2013; Balic et al., 2019). These classifications present a partial correspondence: Luminal A (ER+ and PR+, HER2- and Ki-67 low), luminal B HER2- (ER+, HER2- and at least one of PR negative or low or Ki-67 high), luminal B HER2+ (ER+, HER2+, any Ki-67, and any PR), HER2 enriched (ER-, PR- and HER2+) and basal-like/triple-negative (ER, PR- and, HER2-). The classic biomarkers of immunohistochemical subtypes - estrogen receptor (ER), progesterone receptor (PR), HER2 status, and Ki-67 proliferation index are established factors to determine prognostic and guide the choice of treatment method (Parise and Caggiano, 2014; Fragomeni et al., 2018). However, the clinical application of these biomarkers may be limited once they do not fully reflect tumor heterogeneity (Sun *et al.*, 2019). Thus, the identification of more specific and sensitive biomarkers can lead to relevant clinical implications in individualized patient treatment and the prediction of clinical outcomes (Li et al., 2020).

In this study, we evaluated *in silico* the clinical and functional relevance of the LEF1/TCF family members in breast cancer. We performed bioinformatic analyses and used public databases to investigate the relationship between expression patterns, immune infiltrates, and clinicopathological parameters, including prognostic and biomarker significance. We also explored the biological functions and molecular mechanisms related to these transcription factors, aiming to provide a comprehensive understanding of the relevance of the LEF1/TCF family in breast cancer.

## Materials and Methods

### Differential expression analysis on the GEPIA2 database

GEPIA2 (Tang et al., 2019) is a web server that allows the analysis of mRNA expression data from the TCGA project (Weinstein et al., 2013). We analyzed the expression of *LEF1*, *TCF3*, *TCF4*, and *TCF7* at mRNA levels in 16 cancer types, including breast cancer, comparing the expression of tumor and non-tumor samples (T x NT). This analysis only included cancer types with at least ten non-tumor samples available. The analysis of variance (ANOVA) was performed to access the differential expression in the comparisons (Log^2^FC ±0.58; P-value < 0.05). The same statistical approach was performed to examine the expression of *LEF1*, *TCF3*, *TCF4*, and *TCF7* in the breast cancer molecular subtypes, first applying a T x NT comparison to each subtype separately, and after a comparison between the tumor samples of each subtype (T x T). Also, using the GEPIA2 database, we investigated the mRNA levels of *LEF1*, *TCF3*, *TCF4*, and *TCF7* across different breast tumor stages (P-value <0.05).

Using the TCGA mRNA data and the binary regression model implemented in the IBM SPSS Statistics (v.26) software, we tested the potential of *LEF1*, *TCF3*, *TCF4*, and *TCF7* to discriminate tumor breast samples from non-tumor samples. The performance of each gene was obtained by receiver operating characteristic curves (95% confidence interval; P-values < 0.05), and quantified by the area under de curve (AUC).

### Immunohistochemistry investigation on The Human Protein Atlas (HPA)

The Human Protein Atlas (HPA) (Uhlén et al., 2015) is an online database that uses antibody-based methods to determine the expression of proteins in tumor and non-tumor samples. In this study, we explored the expression of *LEF1* (Antibody: CAB019405), *TCF3* (Antibody: CAB018351), *TCF4* (Antibody: CAB020722), and *TCF7* (Antibody: CAB019402) proteins in tumor and non-tumor breast samples. The protein expression levels were defined based on the staining intensity (not detected, low, medium, or high). We selected the tumor samples with both stronger and weaker staining for comparison with the non-tumor samples. 

### Breast Cancer Gene-Expression and Ualcan database analysis

Bc-GenExMiner (v.4.5) is a statistical tool for mining transcriptomic breast cancer data from DNA microarrays and TCGA samples (Jézéquel et al., 2021). Using the total gene expression data (n= 11,359), we explored the relationship between the expression of *LEF1*, *TCF3*, *TCF4*, and *TCF7* and the breast cancer prognostic factors ER (ER+/ER-), PR (PR+/PR-), HER2 (HER2+/HER2-), nodal status (negative/positive) and patients age (≤51 and >51). TP53 status (mutated/wild-type), PAM50/TNBC status (non-basal/non-TNBC x basal/TNBC), Nottingham Prognostic Index (NPI) and Scarff Bloom & Richardson grade (SBR) were also evaluated (P-value <0.05). Next, Kaplan-Meier survival analyses were performed to evaluate the associations of *LEF1*, *TCF3*, *TCF4*, and *TCF7* with overall survival (OS), distant metastasis-free survival (DMFS), and disease-free survival (DFS) (P-value <0.05). Groups of high and low expression were defined using the median value. Through Bc-GenExMiner, we also investigated the expression of these genes accordingly to the histological subtypes of breast cancer (P-value <0.05).

The methylation status in the promoters of the *LEF1*, *TCF3*, *TCF4*, and *TCF7* genes was determined through UALCAN online tool (Chandrashekar et al., 2017), using the beta-values to determine hyper or hypomethylation on gene promoters in breast tumor compared to non-tumor samples (P-value < 0.05).

### Immune infiltration and immune subtype analysis in TIMER and TISIDB databases

The Tumor Immune Estimation Resource (TIMER) database (Li et al., 2017) is an online tool that allows the analysis of the relation between the immune infiltrates status and gene expression of diverse cancer types. The abundance of six tumor-infiltrating immune cells (B-cells, CD4+ and CD8+ T cells, macrophages, neutrophils, and dendritic cells) were evaluated in breast cancer and correlated to the mRNA expression of *LEF1*, *TCF3*, *TCF4,* and *TCF7* using the database algorithm (correlation of ±0.15; P-value <0.05). Following to the database analysis pipeline, all the correlations were adjusted by tumor purity. 

In the TISIDB web portal (Ru et al., 2019), the expression of *LEF1*, *TCF3*, *TCF4*, and *TCF7* were investigated across the immune subtypes of breast cancer, using the data and subtype classification from TCGA (P-value <0.05).

### Transcription regulatory network and regulon construction

RTN is an R package available in the Bioconductor open-source software (Fletcher et al., 2013; Castro et al., 2015) that tests the association between a given transcription factor (TF) and all potential targets using transcriptomic data. We used RTN (v.2.14.1) to predict transcriptional regulatory networks (TRNs) and determine the regulons (the sets of genes whose expression is influenced by a given TF) related to *LEF1*, *TCF3*, *TCF4*, and *TCF7*. Firstly, we calculated the mutual information (MI) between each TF and all potential targets. Afterward, we applied the MI-based algorithm of the Reconstruction of Accurate Cellular Networks (ARACNe) method (Margolin et al., 2006) to remove non-significant MI values and unstable interactions by permutation and bootstrap, aiming to filtrate the TF-gene pairs and predict the regulons. 

The entire process resulted in consensus regulatory networks, which include a MI value for each TF- gene association combined with a sign (“+” or “-”) that represents the direction of Pearson’s correlation between the pair. The parameters used in the network construction were 1000 permutations, a P-value cutoff of 0.01, and 100 bootstraps. The input data comprised a gene expression matrix originated from the TCGA-BRCA data, containing only the differentially expressed genes identified by GEPIA2 (Log^2^FC ±0.58; P-value < 0.05).

### Molecular signatures database enrichment analysis

The molecular signatures database (MSigDB) (Subramanian et al., 2005) is a web tool composed of a collection of annotated gene sets available for several analyses. We used MSigDB (v.7.4) to perform enrichment analysis on the genes that comprise the regulons of *LEF1*, *TCF3*, *TCF4*, and *TCF7*, aiming to investigate the biological pathways and processes in which these genes take part. Using the global cancer map expression profile, MSigDB computed the overlap between each of the four regulons separately with the REACTOME collection, identifying the top 25 pathways more significantly enriched in the regulons (FDR-value < 0.05).

Results

### The LEF1/TCF family members are differentially expressed in pan-cancer.

**Figure 1 -:**
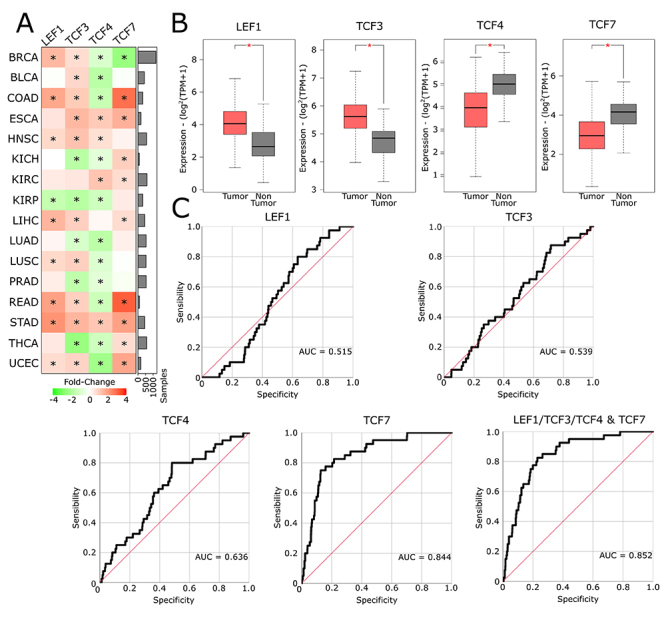
Transcriptional expression levels of LEF1/TCF family members. (A) Heatmap of mRNA expression of *LEF1*, *TCF3*, *TCF4*, and *TCF7* in 16 cancer types, comparing tumor to non-tumor tissues. Red: Over-expression. Green: Down-expression. The bar chart shows the approximate number of samples of each cancer type. (B) Boxplots of the mRNA expression of the LEF1/TCF family members in tumor (red) x non-tumor (grey) breast tissues comparison. (C) Receiver operating curves (ROCs) of breast tumor and non-tumor samples, designed by binary logistic regression models to each gene separately, and associated. AUC = Area under the curve. * = Differential expression at fold-change ± 1.5 (Log2FC ±0.58) and P-value < 0.05.

We used the GEPIA2 database to explore the mRNA levels of the LEF1/TCF transcription factor family members, comparing the differences in their expression between tumor and non-tumor tissue samples of 16 cancer types. These genes were found deregulated in cancer, with expression levels at least 1.5 folds altered in tumor tissues (Figure 1A). *LEF1*, *TCF3*, and *TCF7* were frequently over-expressed in several cancer types, while *TCF4* was commonly down-expressed. More detailed gene expression data are displayed in Table S1. 

### Expression levels, methylation status, and biomarker potential of LEF1/TCF family members in breast cancer subtypes.

In the T x NT breast cancer comparisons, GEPIA2 shows that *LEF1* (Log^2^FC = 1.462, P-value <0.0001) and *TCF3* (Log^2^FC = 0.675, P-value <0.0001) are over-expressed in tumor samples, and *TCF4* (Log^2^FC = -1.028, P-value <0.0001) and *TCF7* (Log^2^FC = -1.210, P-value <0.0001) are down-expressed (Figure 1 B). To determine the biomarker potential of these molecules, we applied binary logistic regression models. As shown in Figure 1 C , *TCF7* (AUC = 0.844) have the most promising discriminative potential do differentiate tumor from non-tumor breast samples, followed by *TCF4* (AUC = 0.636), *TCF3* (AUC = 0.539) and *LEF1* (AUC = 0.515). 

**Figure 2 -:**
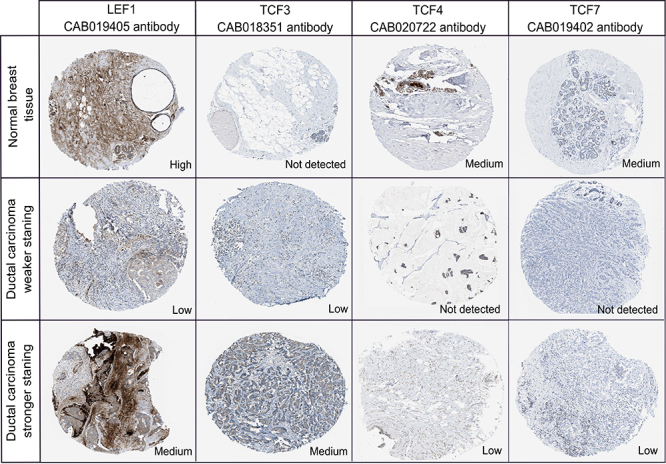
IHC expression pattern of *LEF1*, *TCF3*, *TCF4*, and *TCF7* in breast tumor and non-tumor tissues. Human protein atlas antibody-based IHC of breast non-tumor tissue and tumor breast tissues. To cover the staining spectrum in breast tumors, we compared the non-tumor samples with tumor samples representing the weaker and stronger staining pattern obtained.

Also, to determine if the mRNA expression matches the protein levels, we used the HPA database to analyze the immunohistochemical staining of breast tumor and non-tumor tissues (Figure 2). We found that this antibody-based analysis could detect the protein over-expression of *TCF3* and down-expression of *TCF4* and *TCF7* in breast tumors at levels consistent with that of mRNA. Controversially, *LEF1* showed stronger staining in non-tumor than in the tumor tissue.

**Figure 3 -:**
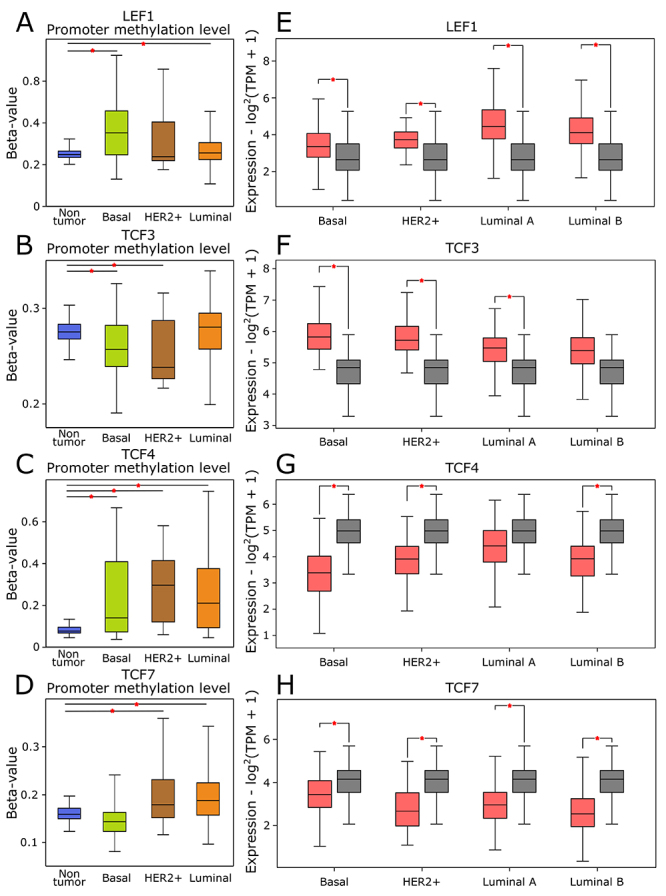
LEF1/TCF family mRNA expression and methylation status in breast cancer molecular subtypes. (A-D) Methylation status on the genes’ promoters, given in beta-values (P-value < 0.05). Blue = Non-tumor samples. Green = Basal-like breast tumors. Brown = HER2+ enriched tumors. Orange = Luminal tumors (luminal A + luminal B). (E-H) Boxplots representing the expression pattern obtained to LEF1, TCF3, TCF4, and TCF7 comparing tumor (red) and non-tumor (grey) tissues subgrouped in basal-like, HER2+ enriched, luminal A and luminal B subtypes (Log2FC ±0.58; P-value < 0.05).

The T vs. NT comparisons were further performed by verifying the methylation status in the gene promoter region (Figure 3 A -D), and subgrouping tumors by molecular subtypes (Figure 3 E  -H). Classically, hypomethylation is related to higher expression, and hypermethylation to gene silencing (Ehrlich, 2009). We observed that *LEF1* was over-expressed in tumors of all the subtypes; controversially, its promoter region was found hypermethylated in basal and luminal tumors. *TCF3* was hypomethylated in basal and HER2 enriched tumors, but not in luminal tumors. Concordantly, *TCF3* showed no significant differential expression in both luminal subtypes but was over-expressed in basal and HER2 enriched tumors. *TCF4* presented no differential expression in luminal A tumors but was down-expressed in the other three subtypes. Regarding methylation, *TCF4* was hypermethylated in all tumor subtypes. *TCF7* presented down-expression in tumors of all subtypes and was hypermethylated in luminal and HER2 enriched tumors. Moreover, Table 1 shows the comparison between tumor samples of each subtype (P-value < 0.05 cutoff).

**Table 1 -:** LEF1, TCF3, TCF4, and TCF7 mRNA expression patterns in subtype comparisons.

*LEF1*		*TCF3*	
Subtype comparison	P-value	Subtype comparison	P-value
Basal like = HER2	P-value > 0.05	Basal like = HER2	P-value > 0.05
Basal like < Luminal A	P-value < 0.05	Basal like > Luminal A	P-value < 0.05
Basal like < Luminal B	P-value < 0.05	Basal like > Luminal B	P-value < 0.05
HER2 < Luminal A	P-value < 0.05	HER2 > Luminal A	P-value < 0.05
HER2 < Luminal B	P-value < 0.05	HER2 > Luminal B	P-value < 0.05
Luminal A > Luminal B	P-value < 0.05	Luminal A = Luminal B	P-value > 0.05
*TCF4*		*TCF7*	
Subtype comparison	P-value	Subtype comparison	P-value
Basal like < HER2	P-value < 0.05	Basal like > HER2	P-value < 0.05
Basal like < Luminal A	P-value < 0.05	Basal like > Luminal A	P-value < 0.05
Basal like < Luminal B	P-value < 0.05	Basal like > Luminal B	P-value < 0.05
HER2 > Luminal A	P-value < 0.05	HER2 = Luminal A	P-value > 0.05
HER2 = Luminal B	P-value > 0.05	HER2 = Luminal B	P-value > 0.05
Luminal A > Luminal B	P-value < 0.05	Luminal A > Luminal B	P-value < 0.05

**Figure 4 -:**
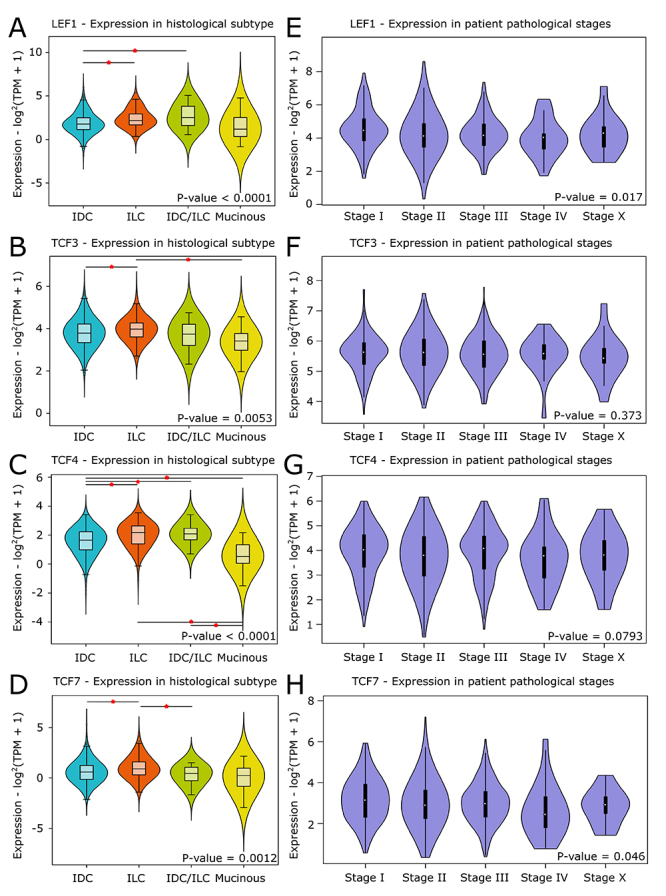
LEF1/TCF family mRNA expression in breast cancer histological types and stages. (A-D) *LEF1*, *TCF3*, *TCF4*, and *TCF7* expression in histological types and (E-H) in different breast cancer stages. IDC: Invasive ductal carcinoma. ILC: Invasive lobular carcinoma. ‘Stage x’ represents tumors whose stage could not be determined.

In addition, the expression of the transcription factors was analyzed regarding the histological types and stages of breast cancer. In general, *LEF1*, *TCF3*, *TCF4,* and *TCF7* presented lightly high expression in invasive lobular carcinoma (ILC) type, while *TCF4* had a lower expression in mucinous type (P-value < 0.05) (Figure 4 A -D). *LEF1* was the only one with a significant association with the tumor stage, presenting higher expression in the initial stages (P-value = 0.017, Figure 4 E ).

### The LEF1/TCF transcription factors are associated to clinicopathological features of breast cancer.

The potential clinical relevance of the LEF1/TCF family in breast cancer was investigated using the statistical mining tool bc-GenExMiner (v.4.5). The mRNA expression levels of *LEF1*, *TCF3*, *TCF4*, and *TCF7* were evaluated according to the five classical breast cancer prognostic factors - ER, PR, and HER2 status, age, and nodal status; the *TP53* status and PAM50/TNBC status were also included in the analysis (Table 2).

**Table 2 -:** Association between LEF1/TCF family expression and prognostic parameters.

	*LEF1*		*TCF3*		*TCF4*		*TCF7*	
	Expression	P-value	Expression	P-value	Expression	P-value	Expression	P-value
**PR status**								
PR+ tumors	Higher expression	< 0.001	Lower Expression	<0.0001	Higher expression	<0.0001	Lower expression	<0.0001
PR- tumors	Lower expression		Higher Expression		Lower expression		Higher expression	
**ER status**								
ER+ tumors	Higher expression	<0.0001	Lower Expression	<0.0001	Higher expression	<0.0001	Lower expression	0.0163
ER- tumors	Lower expression		Higher Expression		Lower expression		Higher expression	
**HER2 status**								
HER2+ tumors	Lower expression	<0.0001	Higher Expression	<0.0001	*	0.3131	Higher expression	0.0002
HER2- tumors	Higher expression		Lower Expression		*		Lower expression	
**PAM50 & TNBC (IHC) classification**								
Non-basal-like & Non-TNBC	Higher expression	<0.0001	Lower Expression	<0.0001	Higher expression	<0.0001	Lower expression	<0.0001
Basal-like & TNBC	Lower expression		Higher Expression		Lower expression		Higher expression	
**Patients age**								
≤ 51 years	*	0.1025	Higher Expression	<0.0001	Higher expression	<0.0001	Higher expression	0.0005
> 51 years	*		Lower Expression		Lower expression		Lower expression	
***TP53* status (IHC)**								
Wild type	Higher expression	0.0245	Lower Expression	<0.0001	Higher expression	0.0029	Lower expression	0.008
Mutated	Lower expression		Higher Expression		Lower expression		Higher expression	
**Lymph node status**								
Positive	*	0.4044	*	0.1255	*	0.6552	*	0.0957
Negative	*		*		*		*	
**Nottingham Prognostic Index status (NPI)**								
	NPI1>NPI2	< 0.01	NPI1=NPI2	0.0716	NPI1 > NPI2	<0.0001	NPI1=NPI2	0.4398
	NPI1>NPI3	< 0.01	NPI1=NPI3		NPI1 > NPI3	<0.0001	NPI1=NPI3	
	NPI2=NPI3	0.1	NPI2=NPI3		NPI2 = NPI3	0.1	NPI2=NPI3	
**Scarff Bloom & Richardson grade status (SBR)**								
	SBR1=SBR2	> 0.1	SBR1=SBR2	>0.1	SBR1>SBR2	<0.0001	SBR1=SBR2	0.10
	SBR1>SBR3	<0.0001	SBR1>SBR3	<0.0001	SBR1>SBR3	<0.0001	SBR3>SBR1	<0.0001
	SBR2>SBR3	<0.0001	SBR2>SBR3	<0.0001	SBR2>SBR3	<0.0001	SBR3>SBR2	<0.0001

* = No significant associations found.

The high expression of *LEF1* was significantly associated with positive ER/PR status and HER2 negative status (P < 0.0001), and *TCF7* had its lower expression associated with ER/PR positive and HER2 negative tumors (P-value < 0.05). In contrast, low expression of *TCF4* was related to negative ER/PR status (P < 0.0001), and higher levels of *TCF3* were associated with negative ER/PR status and HER2 positive status (P-value < 0.0001). Concordantly, *LEF1* and *TCF4* were positively associated with Non-basal-like/Non-TNBC tumors (P-value < 0.0001), while *TCF3* and *TCF7* were positively associated to basal-like/TNBC tumors (P-value < 0.0001). 

The parameters age, *TP53* status and nodal status were also analyzed, highlighting that *LEF1* had a positive correlation with wild type *TP53* tumors (P-value = 0.0245); *TCF3* presented higher levels in ≤ 51 years patients (P < 0.0001) and a positive relation with mutated *TP53* tumors (P-value <0.0001); *TCF4* showed a lower expression in > 51 years patients (P-value < 0.0001) and in *TP53* mutated tumors (P-value = 0.0029), and *TCF7* presented lower expression in > 50 years patients (P-value = 0.0005), and *TP53* wild type tumors (P-value = 0.008).

### *LEF1*, *TCF3*, *TCF4*, and *TCF7* are associated with the prognosis of breast cancer patients 

Considering its associations with clinicopathological and molecular parameters of the disease, together with the possibility that its deregulated expression in breast cancer may impact tumorigenesis, we investigated the potential value of the LEF1/TCF transcription factors as prognostic markers. The prognostic value of *LEF1*, *TCF3*, *TCF4*, and *TCF7* was accessed using bc-GenExMiner (v.4.5), searching for associations between their expression levels and overall survival (OS), disease-free survival (DFS), and distant metastasis-free survival (DMFS).

**Figure 5 -:**
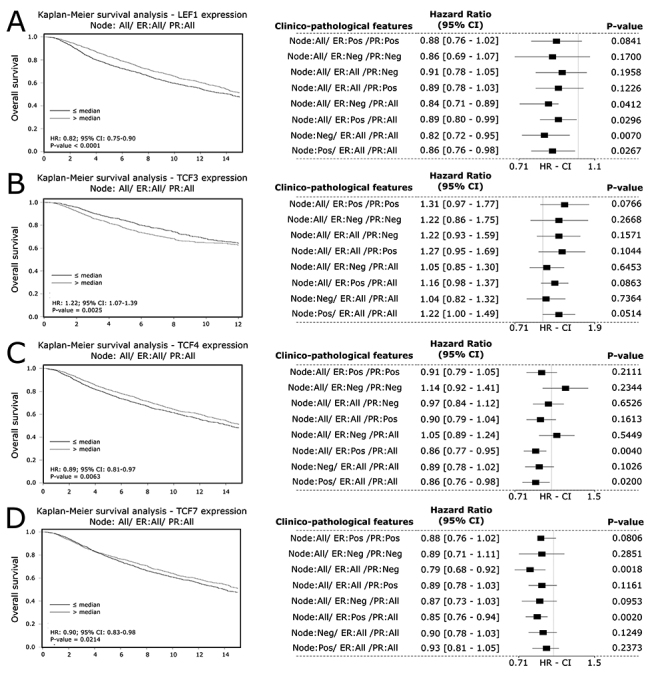
Prognostic value of *LEF1*, *TCF3*, *TCF4*, and *TCF7* in breast cancer patients at mRNA level regarding overall survival. OS associations of (A) *LEF1* (B) *TCF3* and (C) TCF4 and (D) *TCF7*. Forest plots indicate the associations when considering clinicopathological features (P-value < 0.05; 95% CI). CI= Confidence interval. HR = Hazard Ratio.

The Kaplan-Meier analysis revealed that all four mRNAs had significant associations with OS. More specifically, high expression of *LEF1* was associated with a better OS considering all the samples (HR = 0.82, 95% CI 0.75 - 0.90, P-value < 0.001; Figure 5 A ), as well *TCF4* (HR = 0.89, 95% CI 0.81 - 0.97, P-value = 0.0063; Figure 5 C), and *TCF7* (HR = 0.90, 95% CI 0.83 - 0.98, P-value = 0.0214; Figure 5 D ). In contrast, high expression of *TCF3* was associated with poor OS (HR = 1.22, 95% CI 1.07 - 1.39, P-value = 0.0035; Figure 5 B). 

**Figure 6 -:**
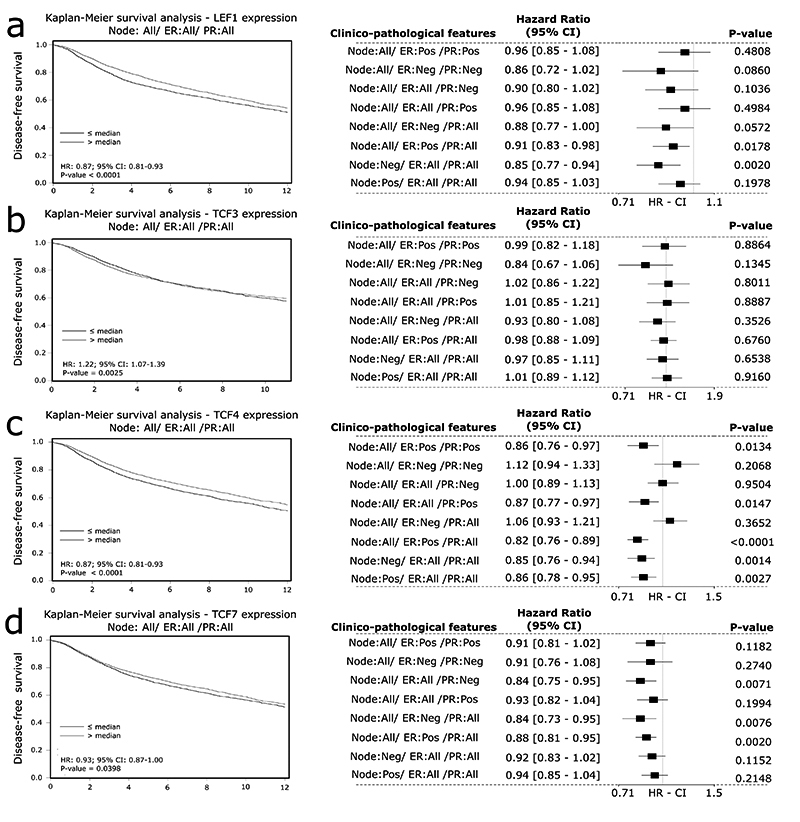
Prognostic value of LEF1, TCF3, TCF4, and TCF7 in breast cancer patients at mRNA level regarding disease-free survival. DFS associations of (A) LEF1 (B) TCF3 and (C) TCF4, and (D) TCF7. Forest plots indicate the associations when samples were subgrouped by clinicopathological features (P-value < 0.05; 95% CI). CI= Confidence interval. HR = Hazard Ratio.

**Figure 7 -:**
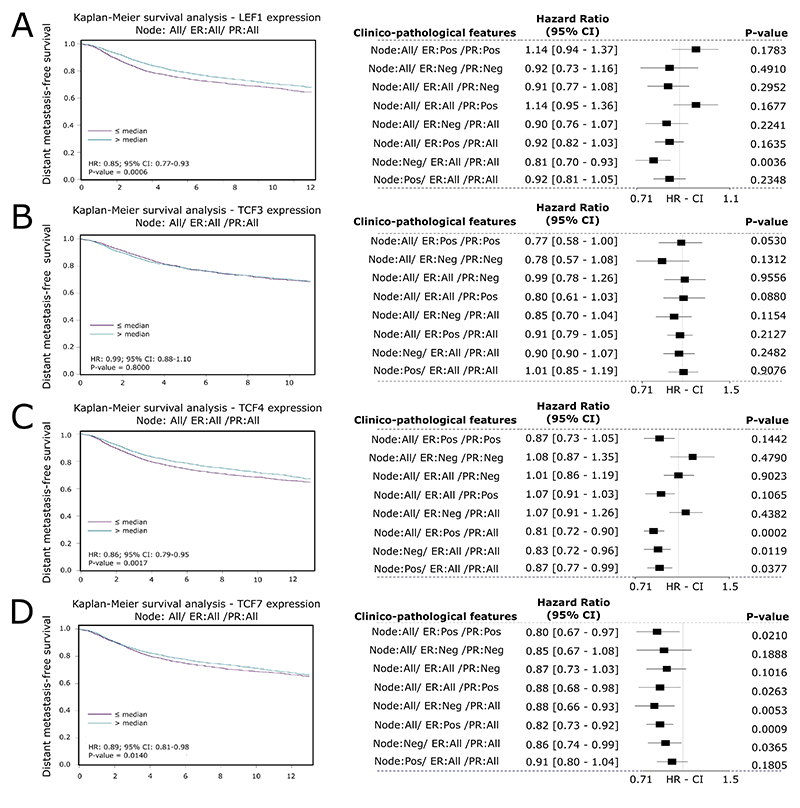
Prognostic value of LEF1, TCF3, TCF4, and TCF7 in breast cancer patients at mRNA level regarding distant metastasis-free survival. DMFS associations of (A) LEF1 (B) TCF3 and (C) TCF4, and (D) TCF7. Forest plots indicate the associations when samples were subgrouped by clinicopathological features (P-value < 0.05; 95% CI). CI= Confidence interval. HR = Hazard Ratio.

*LEF1* high expression was related to a better rate of DFS (HR = 0.87, 95% CI 0.81 - 0.93, P-value < 0.0001; Figure 6 A ), and DMFS (HR = 0.85, 95% CI 0.77 - 0.93, P-value = 0.0006; Figure 7 A ), but *TCF3* expression had no significant association with DFS (Figure 6 B ) or DMFS (Figure 7 B ). *TCF4* low expression was associated with a poor expectation of DFS (HR = 0.87, 95% CI 0.81 - 0.93, P-value < 0.0001; Figure 6 C ) and DMFS (HR = 0.86, 95% CI 0.79 - 0.95, P-value = 0.0017; Figure 7 C ), as well low expression of *TCF7*, which was associated with poor DFS (HR = 0.93, 95% CI 0.87 - 1.00, P-value = 0.0398; Figure 6 D ) and DMFS (HR = 0.89, 95% CI 0.81 - 0.98, P-value = 0.0140; Figure 7 D ).

The forest plots of *LEF1*, *TCF3*, *TCF4*, and *TCF7* related to OS (Figure 5 A  -D), DSF, and DMFS (Figure 6 A -D; Figure 7 A  -D) summarize the associations when the samples were subgrouped by different clinicopathological features. The associations found are concordant with the analysis without subgroups; however, since each subgroup had a low number of samples, it possibly engenders some non-significant P-values.

### *LEF1*, *TCF3*, *TCF4*, and *TCF7* expression influence the presence of immune infiltration markers in breast cancer microenvironment 

We evaluated the correlation between *LEF1*, *TCF3*, *TCF4*, and *TCF7* mRNA levels with six tumor-infiltrating immune cells (B-cells, CD4+ and CD8+ T cells, macrophages, neutrophils, and dendritic cells) using the TIMER database. In addition, we observed their expression pattern in the immunologic subtypes of breast cancer using the TISIDB web source. 

**Figure 8 -:**
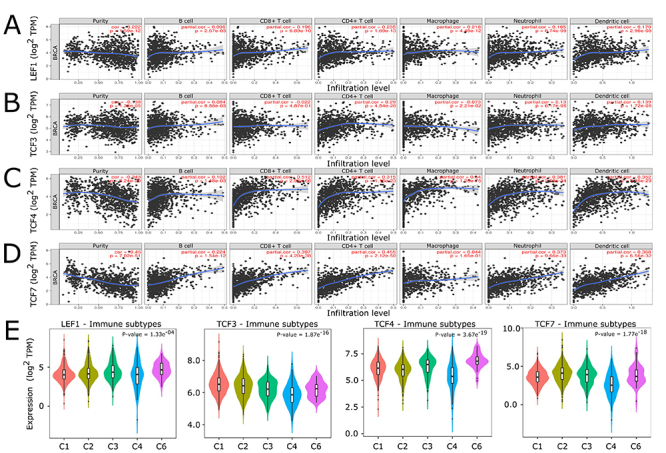
Association between mRNA expression of LEF1, TCF3, TCF4, and TCF7 with tumor infiltration of immune cells and immune breast cancer subtypes. (A-D) TIMER correlations of LEF1, TCF3, TCF4, and TCF7 expression with tumor purity and immune cells. (Correlation of ± 0.15; P-value < 0.05). (E) Expression patterns of LEF1, TCF3, TCF4, and TCF7 across the immune subtypes of breast cancer according to TISIDB. C1: Wound healing. C2: IFN-gamma dominant. C3: Inflammatory. C4: Lymphocyte depleted. C6: TGF-beta dominant.

*LEF1* was related to the infiltration of immune cells, showing a negative association with tumor purity (Cor. = -0.222, P-value < 0.05), and significant-positive associations with five cell markers (Part. cor. > 0.15, P-value < 0.05), except for B-cell infiltrations (Part. cor. = 0.096, P-value < 0.05) (Figure 8 A ). *TCF4*, as like *LEF1*, presented a negative association with tumor purity (Cor. = -0.343, P-value<0.05) and, except for B-cells infiltration (Part. cor. = 0.102, P-value <0.05), presented positive correlations with the other five tumor-infiltrating immune cell markers (Part. cor. > 0.15, P-value < 0.05) (Figure 8 C). *TCF7* was also negatively associated with tumor purity (Cor. = -0.453, P-value<0.05), and positively with tumor infiltration by five immune cells (Part. cor. > 0.15, P-value <0.05), but not with macrophages (Part. cor. = 0.044, P-value = 0.165) (Figure 8 D). In this analysis, *TCF3* only presented a significant-positive relation with the infiltration of CD4+ T cells (Part. cor. = 0.29, P-value <0.05) (Figure 8 B).

The expression levels of *LEF1*, *TCF3*, *TCF4*, and *TCF7* according to different immune subtypes of breast cancer are displayed in Figure 8 E . *LEF1* and *TCF4* were mostly expressed in the inflammatory and TGF-beta dominant subtypes. In contrast, *TCF3* was expressed highly in wound healing and IFN-gamma dominant, and *TCF7* in IFN-gamma in dominant and inflammatory subtypes.

### Regulon’s construction to *LEF1*, *TCF3*, *TCF4,* and *TCF7*

**Figure 9 -:**
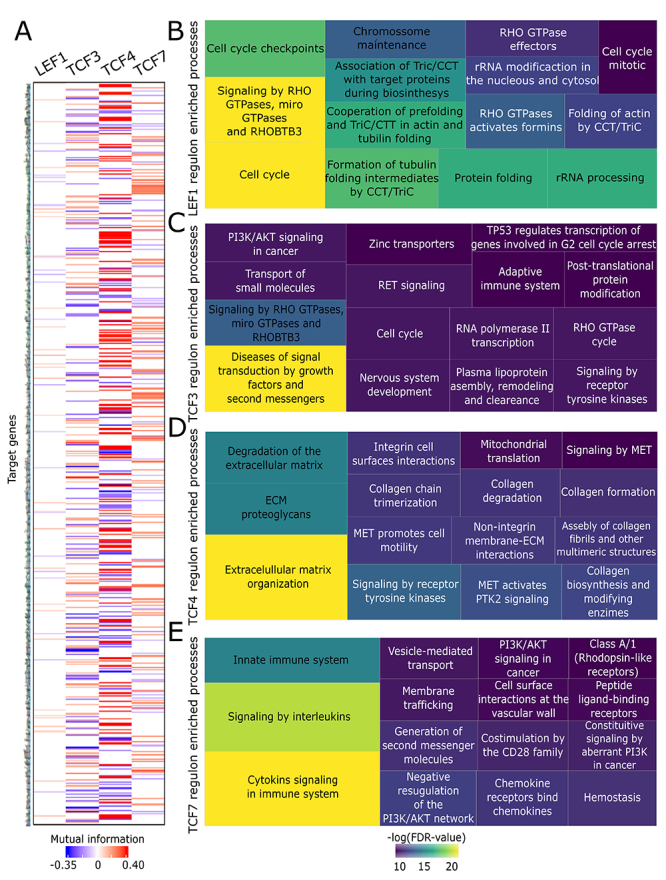
LEF1, TCF3, TCF4, and TCF7 regulon representation and enrichment analysis. (A) Heatmap representation of the final regulon compositions. Red: Higher mutual information (MI) to positive correlations. Blue: Higher mutual information (MI) to negative correlations. (B-E) Treemaps represent the 15 most significantly enriched pathways of each regulon. The size of each box of the treemap is proportional to the number of genes enriched in each pathway (FDR-value < 0.05).

Initially, the RTN analysis resulted in significant TRNs (regulons) composed of *LEF1*, *TCF3*, *TCF4*, and *TCF7* associations with 5269 breast cancer differentially expressed target genes (P-value < 0.01). These genes potentially have its expression influenced by the LEF1/TCF transcription factors. The regulons predicted for *LEF1* and *TCF3*, both over-expressed in breast tumor samples, included 640 and 2421 genes respectively, while the down-expressed *TCF4* and *TCF7* presented 3109 and 2284 genes in its regulons respectively. To retain only the most significant associations for the enrichment analysis, 5% of the most positive and 5% of the negative associations (MI values closer to 1 or -1, respectively) were filtered and maintained. The final regulons included 801 differentially expressed target genes: *LEF1* filtered regulon was composed of 64 genes; the *TCF3* filtered regulon presented 242 genes; *TCF4* retained 311 and *TCF7* 228 genes (Figure 9 A ; Table S2).

### The genes predicted to compose the *LEF1*, *TCF3*, *TCF4*, and *TCF7* regulons participate in processes and pathways involved in breast cancer tumorigenesis 

The MSigDB analysis showed that the genes present in the regulons were significantly enriched in pathways and biological functions associated with carcinogenesis (FDR q-value < 0.05) (Table S3). Figure 9 B-E displays the 15 most significant enrichments of each regulon. 

The *LEF1* regulon was mainly associated with cell cycle regulation, RHO GTPase signaling, chromosome maintenance, and processes related to the CCT/TriC chaperonins functions (Figure 9 B). The *TCF3* regulon contained genes involved in signal transduction, including signaling by receptors tyrosine kinase, PI3K/AKT signaling, and RET signaling (Figure 9 C ). The genes present in *TCF4* regulon showed a close relation to extracellular matrix (ECM), including degradation and organization of ECM, collagen degradation and trimerization, and MET signaling (Figure 9 D ). The *TCF7* regulon was enriched mainly with immune system processes, like cytokine signaling, innate immune system, and chemokine receptors, as well as PI3K/AKT signaling and network (Figure 9 E ).

## Discussion

Breast cancer continues to require attention due to its crescent incidence and high mortality rate in women worldwide. Although molecular biology and bioinformatics have improved the clinical research, new biomarkers of prognostic, diagnostic, and therapeutic targets are still needed to reinforce and complement the classic breast cancer prognostic factors ER, PR, HER2, age, and lymph node status (Laila et al., 2019; Yu et al., 2019; Gong et al., 2020). In this study, we used bioinformatic analysis to perform an in-depth investigation of the expression pattern and clinicopathological associations of the LEF1/TCF family members in breast cancer. 

A pan-cancer view revealed that *LEF1*, *TCF3*, *TCF4*, and *TCF7* have aberrant expression and are potentially involvement in the tumorigenesis of various cancer types. The direction of the dysregulation of these gene expression (down-/over-expression), however, varied greatly between cancer types, indicating a possible tissue-dependent tumorigenic action. Regarding the biomarker potential in breast cancer, our results suggest that LEF1, TCF3, TCF4, and specially TCF7, have significant diagnostic value to distinguish breast cancer patients from healthy individuals and a role in subtyping insight. 

Previous studies demonstrated an association between higher expression of *LEF1* with the expression of ER/PR and activation of the Wnt pathway in luminal subtypes, as well as a negative correlation between *LEF1* and HER2 expression, indicating that *LEF1* tends to mediate tumor cell invasion mainly in tumors positives to ER/PR and lacking HER2 over-expression (Nguyen et al., 2005; Lim et al., 2011; Lamb et al., 2013). Likewise, we found over-expression of *LEF1* in tumor tissues of all subtypes, but especially in luminal (ER+/PR+/HER2-) tumors. *TCF3* also appears over-expressed in breast tumor tissues, but when subgrouping tumors by subtypes, *TCF3* showed higher expression only in basal and HER2 enriched subtypes, corroborating previous observations of over-expression of *TCF3* in ER- tumors and its association with basal-like tumors (Slyper et al., 2012; Zheng et al., 2019). 

*TCF4*, appointed as a tumor suppressor in breast cancer (Shulewitz et al., 2006), was down-expressed in tumor samples, especially in non-luminal subtypes (ER-/PR-). This suggests that the loss of this tumor suppressor can be involved in the aggressive behavior of HER2 enriched and basal subtypes. Among the analyzed cancer types, breast cancer was the only one to present a down-expression of *TCF7*; no studies have previously appointed its low expression in breast tumors or analyzed the functional impacts decurrent of a loss of expression. Searching for the methylation status at the promoter region of the LEF/TCF genes in tumor and non-tumor samples, we found a fair correspondence between methylation status and mRNA expression, indicating a possible origin for its dysregulated expression in malignant breast tissues. 

Once confirmed the aberrant expression of these molecules in breast cancer, we addressed their potential as prognostic markers through Kaplan-Meier analysis of OS, DFS, and DMFS. High expression of *LEF1* was previously correlated with poor prognosis in several cancer types, like oral squamous cell carcinoma (Su et al., 2014), nasopharyngeal carcinoma (Zhan et al., 2019), and lung cancer (Bleckmann et al., 2013), however, as observed in colorectal cancer (Kriegl et al., 2010), our survival analysis indicated *LEF1* low expression to be significantly associated with poor OS, DFS, and DMSF rates. Interestingly, *LEF1* had a lower expression in HER2 enriched and basal-like, the more aggressive subtypes. *TCF4* low expression was also significantly associated with poor OS, DFS, and DMSF rates, corroborating previous observations that breast cancer patients with higher expression of *TCF4* have a better prognosis, also supporting the hypothesis that *TCF4* may have tumor suppressor activities in breast cancer (Ravindranath et al., 2011). *TCF7* also had its low expression associated with poor prognosis, suggesting that hypermethylation and low expression of this transcription factor could represent the loss of a tumor suppressor in breast cancer. *TCF3* over-expression, in turn, was associated with poor OS in our analysis, like in nasopharyngeal carcinoma (Shen et al., 2017) and colorectal cancer (Li et al., 2014). Concerning the commonly accepted prognostic factors NPI and SBR, our results demonstrated that advanced NPI and SBR grades go along with low mRNA expression of *LEF1* and *TCF4*, corroborating the Kaplan-Meier results. As for *TCF3*, we found an increased expression in lower NPI grades, but no significant association was found with SBR grades, while *TCF7* was not associated with NPI but with advanced SBR grades.

Further, we considered the well-known involvement of the LEF1/TCF family with the lymphatic and immune system to investigate its implication in immunologic subtypes and the abundance of immune infiltrates in breast cancer. It has been reported that *LEF1*, *TCF4,* and *TCF7* are involved in the maturation and malignant transformation of thymocytes, development of natural killer and T cells, and through Wnt pathway, tumor infiltration and immune evasion (Yu et al., 2012; Haseeb et al., 2019; Crispin and Tsokos, 2020). In breast cancer, tumor immune infiltration is clinically relevant to predicting outcomes: The composition and abundance of immune cells can serve as biomarkers for survival and treatment response in terms of chemotherapy and immunotherapy (Oshi et al., 2021).

Immune cells can significantly influence the tumor microenvironment and growth through anti-tumor immunity, cell-mediated cytotoxicity, inflammation, and secretion of cytokines and growth factors (Goff and Danforth, 2021). In breast cancer, high expression of CD4+ and CD8+ T cells (Lacko et al., 2008) and the accumulation of tumor-associated macrophages (Weinstein et al., 2013), dendritic cells (Szpor et al., 2021) and neutrophils (Wculek and Malanchi, 2015) were associated with prognosis, although there are disagreements about whether they are related to favorable or unfavorable prognosis (Mahmoud et al., 2011; Stanton and Disis, 2016). Our analysis shows that *LEF1* has a more accentuated down-expression in the breast cancer immune subtypes with less favorable outcomes (wound healing and IFN-gamma dominant subtypes), while *TCF4* and *TCF7* were mainly down-expressed in the lymphocyte depleted subtype, a subtype with mixed signatures (Thorsson et al., 2018). A negative correlation with tumor purity and a positive correlation with the presence of CD4+ and CD8+ T cells, macrophages, neutrophils, and dendritic cells was observed in these three transcription factors, implying the over-expression of *LEF1* in augmentation of the levels of immune infiltrating cells in breast microenvironment, and low expression of *TCF4* and *TCF7* to ablation of immune cells infiltration. *TCF3* was highly expressed in wound healing and IFN-gamma dominant subtypes, but with a non-significant correlation with tumor purity. Together, these results suggest a relevant role of *LEF1*, *TCF4*, and *TCF7* in the immune tumor microenvironment of breast cancer and support their application as prognosis markers. 

Finally, we investigated the potential role of these transcription factors on breast tumorigenesis by determining its regulons, and the processes and pathways in which they are involved. Our analysis showed that the regulon of *LEF1* was mainly associated with pathways related to cell cycle regulation, Rho GTPases signaling, and metastasis induction through CCT/TriC chaperonins. These findings support previous reports on the *LEF1* function in cancer malignancy: In colon cancer, for example, knockdown of *LEF1* reduced cell viability, invasion capacity, and proliferation through cell cycle stabilization (Wang et al., 2013). In prostate cancer, *LEF1* is involved in cell cycle regulation, proliferation, and metastasis (Liang et al., 2015), and in bladder cancer, related to epithelial-to-mesenchymal transition (EMT) induction (Xie et al., 2020). In breast cancer, *LEF1* acts in metastatic processes (Nguyen et al., 2005) and is one of the few commonly over-expressed genes in brain-seeking breast cells (Blazquez et al., 2020). Reportedly, over-expression of *LEF1* leads to deregulation of several pathways, contributing to tumorigenic processes. However, as a prognosis marker, it is low expression of *LEF1* that is associated with poor prognosis in breast cancer: This conflict may be the result of the interaction patterns or changes in the tumor microenvironment that are yet to be unraveled.

In several cancer types, *TCF3* over-expression is associated with tumorigenic processes. In colorectal and gastric cancer, *TCF3* is related to proliferation stimulation and metastasis (Li et al., 2014; Taniue et al., 2016; Zhang et al., 2019), and in skin cancer, *TCF3* knockdown decreased tumor growth and aggressiveness (Ku et al., 2017). In breast cancer, *TCF3* is linked with tumor growth and initiation (Slyper et al., 2012), and in the triple-negative/basal subtype, *TCF3* was related to proliferation, migration, and apoptosis (Jia et al., 2020). Our results appoint to the participation of *TCF3* regulon in cell cycle regulation, Rho GTPases cycle, adaptive immune system, RET signaling, PI3K/AKT signaling, besides signal transduction by growth factor receptors and tyrosine-kinase receptors.

*TCF4* is known as a tumor suppressor in some cancer types: In colon cancer, loss of *TCF4* leads to tumorigenesis via dysregulation of proliferation (Angus-Hill et al., 2011) and metastasis (Anwar et al., 2020), and in medulloblastoma, in vitro over-expression of *TCF4* suppressed cell proliferation and growth (Hellwig et al., 2019). In breast cancer, *TCF4* is also suggested to play a role in tumor suppression (Shulewitz et al., 2006; Ravindranath et al., 2011), with low expression of *TCF4* being related to chemoresistance in breast cancer xenograft models via cell cycle deregulation (Ruiz de Garibay et al., 2018) and to metastasis, having its low expression accentuated in breast-to-brain metastasis (Mamoor, 2021). Our enrichment analysis associated the *TCF4* regulon mainly with metastasis-related processes, like extracellular matrix organization, degradation and proteoglycans, cell surface integrin interactions, and collagen biosynthesis and degradation via regulation of collagen genes. Altogether, our results reinforce that low expression of *TCF4* contributes to breast cancer malignancy. 

*TCF7* regulon was mainly enriched in processes involving the immune system, cytokine signaling, chemokine receptors, and PI3K/AKT signaling. The down-expression of *TCF7* is rarely related to cancer, however, it has been demonstrated that depletion of *TCF7* can impact immune system regulation and immunotherapy response (van der Leun et al., 2020). *TCF7* also participates in chemokine signaling in several cancer types (Zhang et al., 2020), highlighting the relevance of this transcription factor in the immune microenvironment and immune signaling of breast tumors. 

In summary, we suggest that *LEF1*, *TCF3*, *TCF4*, and *TCF7* have the potential to be biomarkers in breast cancer clinics. Our study appoints these transcription factors as differentially expressed in breast tumor samples, and that its expression can be related to outcome prediction, immunological subtypes, and immune infiltration in the breast tumor microenvironment. Regarding biological significance, our analysis showed that these transcription factors and their targets are involved in breast tumorigenesis, mainly through cell cycle regulation, metastatic processes, and immune system regulation. This study contributes with relevant data in biomarker discovery and diagnosis/prognosis refinement, suggesting biomarkers that can complement the classic breast cancer prognostic factors. 

## Supplementary material

The following online material is available for this article:

Table S1 - mRNA expression of LEF1, TCF3, TCF4, and TCF7 in 16 cancer types.

Table S2 - Regulon’s composition of LEF1, TCF3, TCF4, and TCF7.

Table S3 - Regulon pathway analysis. (A) LEF1, (B) TCF3, (C) TCF4, and (D) TCF7.
