# Supplementary material for: Biomarker potential of the LEF1/TCF family members in breast cancer: Bioinformatic investigation on expression and clinical significance
Source: Genet Mol Biol. 2023 Dec 15;46(4):e20220346. doi: 10.1590/1678-4685-GMB-2022-0346 (PMC10723634; doi:10.1590/1678-4685-GMB-2022-0346)
Supplement: Table S1 - [file 1415-4757-GMB-46-4-e20220346-s1.pdf]

Genetics and Molecular Biology
https://doi.org/10.1590/1678-4685-GMB-2022-0346

Supplementary Material to “Biomarker potential of the LEF1/TCF family members in breast cancer: Bioinformatic investigation on expression and clinical significance”

Table S1 – mRNA expression of LEF1, TCF3, TCF4, and TCF7 in 16 cancer types compared to non-tumor samples. The number of tumor and non-tumor samples per cancer type is also provided.

	LEF1		TCF3		TCF4		TCF7			
	Log^{2}FC	P-value	Log^{2}FC	P-value	Log^{2}FC	P-value	Log^{2}FC	P-value	TUMOR SAMPLES (N)	NORMAL SAMPLES (N)
BRCA (Breast invasive carcinoma)	1.462	1.01E-64	0.675	5.84E-49	-1.028	1.80E-33	-1.21	8.57E-07	1085	112
BLCA (Bladder Urothelial Carcinoma)	-0.062	4.28E-01	0.828	1.21E-11	-1.768	5.89E-09	-0.091	8.22E-01	404	19
COAD (Colon adenocarcinoma)	1.786	1.18E-73	1.069	1.08E-75	-1.475	2.85E-30	2.829	1.72E-138	275	41
ESCA (Esophageal carcinoma)	0.537	2.05E-14	1.448	9.46E-61	1.103	1.13E-24	1.603	3.52E-42	182	13
HNSC (Head and Neck squamous cell carcinoma)	0.799	1.03E-04	1.251	1.33E-31	0.626	4.54E-10	0.232	0.232	519	44
KICH (Kidney Chromophobe)	0.922	2.99E-01	-1.764	1.42E-10	-0.716	1.22E-02	1.008	2.96E-03	66	25
KIRC (Kidney renal clear cell carcinoma)	0.416	5.41E-05	0.477	9.02E-17	1.199	1.16E-15	0.742	2.54E-12	523	72
KIRP (Kidney renal papillary cell carcinoma)	-1.545	1.67E-13	-1.811	4.82E-27	-1.237	1.76E-07	0.371	1.12E-01	286	32
LIHC (Liver hepatocellular carcinoma)	1.508	8.05E-29	0.952	4.45E-35	0.144	3.12E-02	0.709	1.80E-11	396	50
LUAD (Lung adenocarcinoma)	0.162	5.94E-16	-1.06	6.11E-34	-1.742	5.47E-109	0.286	2.69E-02	483	59

LUSC (Lung squamous cell carcinoma)	0.806	1.72E-17	0.966	2.68E-67	-0.968	7.58E-44	-0.027	9.00E-02	486	50
PRAD (Prostate adenocarcinoma)	0.33	1.30E-02	-1.636	1.32E-62	-0.787	3.14E-07	-0.214	3.87E-01	492	52
READ (Rectum adenocarcinoma)	1.935	1.04E-44	0.973	3.48E-33	-1.342	5.83E-13	3.132	1.02E-94	92	10
STAD (Stomach adenocarcinoma)	2.173	1.08E-79	1.508	9.72E-63	1.155	1.52E-19	1.937	1.05E-59	408	36
THCA (Thyroid carcinoma)	0.439	6.88E-04	-2.462	3.30E-123	-1.236	2.14E-75	0.729	1.10E-06	512	59
UCEC (Uterine Corpus Endometrial Carcinoma)	0.735	8.44E-03	0.864	8.13E-12	-2.312	5.53E-31	1.794	1.79E-16	174	13
