# Supplementary material for: Biomarker potential of the LEF1/TCF family members in breast cancer: Bioinformatic investigation on expression and clinical significance
Source: Genet Mol Biol. 2023 Dec 15;46(4):e20220346. doi: 10.1590/1678-4685-GMB-2022-0346 (PMC10723634; doi:10.1590/1678-4685-GMB-2022-0346)
Supplement: Table S2 - [file 1415-4757-GMB-46-4-e20220346-s2.pdf]

Genetics and Molecular Biology
https://doi.org/10.1590/1678-4685-GMB-2022-0346

Supplementary Material to "Biomarker potential of the LEF1/TCF family members in breast cancer: Bioinformatic investigation on expression and clinical significance"

Table S2 – Regulon's composition of *LEF1*, *TCF3*, *TCF4*, and *TCF7*. The values refer to the mutual information in the TF-gene pairs.

Genes	*LEF1*	*TCF3*	*TCF4*	*TCF7*
AAGAB	0	0	0	-0.06789
ABAT	0.043965	0	0	0
ABCA6	0	0	0.266047	0
ABCA9	0	0	0.212046	0
ABCB1	0	0	0	0.096535
ABCC9	0	0	0.236626	0
ABCD2	0	0	0	0.111611
ABRACL	0	0.069202	0	0
ACER2	0	-0.11005	0	0
ACLY	0	0	0	-0.06618
ADAM12	0	0	0.295477	0
ADAMTS5	0	0	0.182607	0
ADGRA2	0	0	0.225514	0
ADGRF5	0	0	0.188464	0
ADIPOR1	0	0	0	-0.09533
ADM5	0	0.086517	0	0
AFF3	0	-0.11186	0	0
AFTPH	0	-0.12942	0	-0.06903
AGGF1	0	-0.15806	0	0
AGR3	0.046096	0	0	0
AIFM1	0	0	-0.12158	0
AKAP12	0	0	0.302435	0
AKNA	0	0	0	0.161078
ALG3	0	0	-0.12788	0
ALYREF	0	0.079249	0	0
ANAPC11	0	0	-0.12474	0
ANGPTL1	0	0	0.180521	0
ANGPTL2	0	0	0.252644	0
ANK2	0	0	0.183918	0
ANKEF1	0	-0.10805	0	0
ANTXR2	0	0	0.256738	0
AP1M2	0	0	-0.12147	-0.08608
AP1S1	0	0	-0.12443	0
APOO	0	0	-0.13155	0
ARF3	0	-0.10423	0	-0.07467
ARF5	0	0.07998	0	0

ARFGEF1	0	-0.10875	0	0
ARFGEF3	0	0	0	-0.08435
ARHGAP20	0	0	0.284796	0
ARHGAP24	0	0	0.207353	0
ARHGAP31	0	0	0.208017	0
ARHGAP6	0	0	0.202094	0
ARHGDIB	0.049211	0	0	0
ARHGEF1	0	0.100825	0	0
ARID4A	0	-0.11102	0	0
ARL1	0	-0.16446	0	-0.09301
ARMC6	0	0.085319	-0.13764	0
ARMT1	0	-0.1141	0	-0.07636
ARPC1A	0	0	-0.12921	0
ASPN	0	0	0.208425	0
ATAD3B	0	0.106043	0	0
ATP6AP1	0	0	0	-0.06783
ATP6V0B	0	0	-0.1442	0
ATP6V1A	0	-0.16809	0	0
ATP8B1	0	-0.12679	0	0
AXL	0	0	0.246178	0
B4GALT2	0	0.104695	0	0
BACH2	0	0	0.195655	0.141421
BAIAP2L2	0	0.074747	0	0
BANK1	0	0	0	0.132928
BAX	0	0.104096	0	0
BBOX1	0	0	0	0.107199
BCAS1	0	-0.10672	0	-0.07195
BCDIN3D	0	0	0	-0.06812
BCL11A	0	0	0	0.143854
BCL2L12	0	0.153635	0	0
BEND3	-0.04374	0	0	0
BICC1	0	0	0.332005	0
BMP2	0	0	0	0.096294
BNC2	0	0	0.374315	0
BOC	0	0	0	0.124695
BOLA1	0	0	-0.15787	0
BOP1	0	0.077907	0	0
BTRC	0	-0.11039	0	0
BYSL	-0.07402	0	-0.11957	0
C1orf43	0	0	0	-0.08883
C1R	0	0	0	0.099535
C21orf58	0	0	-0.12805	0
C2orf88	0	0	0	0.109849
C3	0	0	0	0.102965
C8orf88	0	0	0.203464	0
C9orf64	0	-0.11949	0	0

CACNA2D1	0	0	0.190421	0
CADM3	0	0	0	0.121993
CALCOCO2	0	-0.16939	0	0
CALD1	0	0	0.213567	0
CANT1	0	0	0	-0.09015
CAPN10	0	0.076866	0	0
CAPN6	0	0	0	0.117815
CCDC107	0	0.09229	0	0
CCDC117	0	-0.11763	0	0
CCDC130	0	0.088745	0	0
CCDC146	0.041265	0	0	0
CCDC167	0	0	-0.1264	0
CCDC69	0	0	0	0.104078
CCDC80	0	0	0.302597	0
CCDC82	0	0	0	0.099935
CCDC85B	0	0.079715	0	0
CCL21	0	0	0	0.103916
CCND2	0	0	0.211501	0
CCNE1	-0.04617	0	0	0
CCR4	0	0	0	0.105441
CCT5	-0.0559	0	0	0
CCT6A	-0.0548	0	0	0
CCT7	0	0	-0.148	0
CD1E	0	0	0	0.12007
CD2	0	0	0	0.127235
CD24	-0.05035	0	0	0
CD27	0	0	0	0.137808
CD302	0	-0.10904	0	0
CD37	0	0	0	0.124496
CD3D	0	0	0	0.138871
CD3E	0	0	0	0.147236
CD3G	0	0	0	0.135063
CD40	0	0	0	0.121209
CD48	0	0	0	0.127284
CD5	0	0	0	0.151086
CD52	0	0	0	0.122184
CD6	0	0	0	0.13684
CD79A	0	0	0	0.115592
CD79B	0	0	0	0.148203
CD93	0	0	0.204272	0
CDC20	0	0.07712	0	0
CDC37	0	0.11504	0	0
CDCA4	0	0.086642	0	0
CDH11	0	0	0.291609	0
CDK5	0	0	-0.14401	0
CDS1	0	0	0	-0.07578

CDT1	0	0.086	-0.1658	0
CEBPB	0	0.068223	0	0
CELF2	0	0	0	0.122984
CENPM	0	0	-0.15965	0
CENPN	-0.0498	0	0	0
CENPO	-0.04796	0	0	0
CENPT	0	0.086156	0	0
CENPW	0	0.08566	0	0
CEP126	0.05756	0	0	0
CERKL	0.073658	0	0	0
CERS2	0	0	0	-0.09569
CERS6	0	-0.17149	0	-0.07343
CFAP69	0.040679	0	0	0
CFH	0	0	0.28927	0
CFL1	0	0.072154	0	0
CFP	0	0	0	0.139227
CH25H	0	0	0	0.106642
CHAD	0.042355	0	0	0
CHAF1A	0	0.106938	-0.15036	0
CHCHD2	0	0	-0.12114	0
CHD9	0	-0.10892	0	0
CHI3L1	0	0	0	0.111604
CHST2	0	0	0	0.124514
CLASRP	0	0.088419	0	0
CLCN3	0	-0.13342	0	0
CLDN15	0	0.072086	0	0
CLDN7	0	0	-0.12227	0
CLEC10A	0	0	0	0.146037
CLIC2	0	0	0	0.110985
CLMP	0	0	0.20212	0
CLTC	0	-0.10765	0	0
CMBL	0	0	0	-0.09716
CMTM7	0	0	0	0.110957
CNRIP1	0	0	0.275956	0
CNTN1	0	0	0.202369	0
COA7	-0.04631	0	0	0
COL12A1	0	0	0.197224	0
COL14A1	0	0	0.199746	0
COL15A1	0	0	0.200002	0
COL1A1	0	0	0.189695	0
COL1A2	0	0	0.238022	0
COL3A1	0	0	0.276261	0
COL4A3	0	0	0	0.102587
COL5A1	0	0	0.204538	0
COL5A2	0	0	0.275749	0
COL8A1	0	0	0.210084	0

COPE	0	0	-0.12183	0
COPZ1	0	0	0	-0.09602
CORO1A	0	0	0	0.111787
CORO2B	0	0	0.192222	0
CPED1	0	0	0.244464	0
CPNE3	0	-0.14289	0	0
CREB3L4	0	0	0	-0.07178
CREBRF	0	-0.16442	0	0
CRISPLD2	0	0	0.258153	0
CRNKL1	0	0	0	-0.0845
CROCC	0	0.076014	0	0
CSK	0	0.095125	0	0
CSNK1A1	0	-0.13736	0	0
CST2	0.040222	0	0	0
CTPS1	-0.04329	0	0	0
CTR9	0	-0.11529	0	0
CTXN1	0	0.106123	0	0
CX3CL1	0	0	0	0.114932
CXCL1	0	0	0	0.110076
CXCL12	0	0	0.230015	0
CXCL2	0	0	0	0.104154
CXCR3	0	0	0	0.119265
CXCR6	0	0	0	0.122945
CXXC1	0	0.085699	0	0
CYB561	0	0	0	-0.07814
CYB5R1	0	0	0	-0.06892
CYC1	0	0	-0.14194	0
DAB2	0	0	0.264744	0
DCAF13	-0.05119	0	0	0
DCHS1	0	0	0.181934	0
DCN	0	0	0.314468	0
DDIT4	0	0.076346	0	0
DDR2	0	0	0.267113	0
DKC1	-0.07778	0	0	0
DLG3	0	0	0	-0.07758
DNAL1	0	-0.13896	0	0
DNASE1L3	0	0	0	0.100023
DNMT3A	0	0.068595	0	0
DOCK11	0	0	0.205152	0
DPP4	0	0	0.209252	0
DPY30	0	0	-0.15245	0
DSCC1	-0.04938	0	0	0
DSE	0	0	0.220616	0
DTYMK	0	0.075199	-0.13468	0
DUS3L	0	0.123337	0	0
E2F1	0	0	-0.16675	0

E2F4	0	0.073491	0	0
EAF1	0	-0.13608	0	-0.06312
EBF1	0	0	0.246768	0
EBNA1BP2	0	0	-0.18426	0
EBP	0	0	-0.13271	0
ECM2	0	0	0.343286	0
EDEM3	0	-0.13911	0	0
EFNA4	0	0.109687	0	0
EFR3A	0	-0.13377	0	0
EGFR	0	0	0	0.097663
EIF5A	0	0	-0.14369	0
EIF6	0	0	-0.12498	0
ELAVL1	0	0	-0.13517	0
ELMOD2	0	0	0	-0.08642
EPB41L5	0	-0.11938	0	0
EPN3	0	0	0	-0.06969
ERBB3	0	0	0	-0.08018
ERG	0	0	0.192313	0
ERGIC1	0	0	0	-0.06316
ERI2	0	-0.10914	0	-0.07691
ERLEC1	0	-0.1682	0	0
ESR1	0	-0.14613	0	-0.09156
ESRP1	-0.05587	0	0	0
ETS1	0	0	0	0.108108
EVL	0.041022	0	0	0
EXOSC4	0	0	-0.15893	0
EXOSC5	0	0	-0.16312	0
F13A1	0	0	0.241503	0
F2R	0	0	0.250338	0
F7	0.05282	0	0	0
FAF2	0	-0.11518	0	0
FAM102B	0	-0.10619	0	0
FAM120A	0	-0.11789	0	0
FAM171A1	0	0	0	0.097449
FAM20A	0	0	0	0.123056
FAM83D	-0.04353	0	0	0
FAM89B	0	0.092882	0	0
FAP	0	0	0.292915	0
FARSA	0	0	-0.19798	0
FAS	0	0	0	0.124801
FAT2	0	0	0	0.101083
FAT4	0	0	0.28675	0
FBXL19	0	0.13278	0	0
FCER2	0	0	0	0.13687
FCMR	0.043573	0	0	0
FCN1	0	0	0	0.121986

FDCSP	0	0	0	0.14574
FERMT2	0	0	0.213547	0
FGD2	0	0	0	0.111295
FGD3	0.057432	0	0	0
FGF7	0	0	0.208363	0
FGR	0	0	0	0.106044
FHL1	0	0	0.185963	0
FIBIN	0	0	0.194527	0
FILIP1L	0	0	0.261828	0
FKBP4	0	0	0	-0.06806
FLAD1	0	0	-0.14866	0
FLI1	0	0	0.180045	0.114182
FLNC	0	0	0.179477	0
FLRT2	0	0	0.288412	0
FOXA1	0	0	0	-0.15523
FOXN3	0	0	0.199372	0
FOXO1	0	0	0.246442	0
FOXP4	0	0.073026	0	0
FREM1	0	0	0	0.099069
FRS2	0	-0.15589	0	0
FRY	0	-0.12546	0	0
FSTL1	0	0	0.312338	0
FUCA1	0.044459	0	0	0
FXYD2	0	0	0	0.130198
FYN	0	0	0	0.131962
GAB3	0	0	0	0.114236
GALNT15	0	0	0.224055	0
GALNT7	0	0	0	-0.07259
GAPT	0.084805	0	0	0
GAS7	0	0	0.283615	0
GATA3	0	0	0	-0.07458
GIMAP5	0	0	0	0.115698
GIMAP7	0	0	0	0.111858
GLIPR2	0	0.072512	0	0
GLT8D2	0	0	0.274908	0
GNG2	0	0	0.247904	0
GOLGA5	0	0	0	-0.06759
GPD2	0	-0.14582	0	0
GPR132	0.042686	0	0	0
GREM2	0	0	0.185357	0
GRID1	0	0	0.182891	0
GRPR	0.048097	0	0	0
GSK3A	0	0	-0.11965	0
GXYLT2	0	0	0.270394	0
GYPC	0	0	0	0.10441
GZMA	0	0	0	0.116535

GZMK	0	0	0	0.129081
HACD3	0	-0.11886	0	-0.07896
HAPLN3	0	0.093177	0	0.12642
HAX1	0	0	-0.1528	0
HCFC2	0	-0.18806	0	0
HES4	0	0.089867	0	0
HGF	0	0	0.246815	0
HHEX	0.081719	0	0	0
HID1	0	0	0	-0.09873
HIPK3	0	-0.15664	0	0
HMBS	0	0	-0.12853	0
HR	0	0.071604	0	0
HSD17B10	0	0	-0.17246	0
HSPA9	0	0	0	-0.06334
HSPD1	-0.08227	0	0	0
HSPE1	0	0	-0.125	0
HSPG2	0	0	0.20718	0
IARS2	0	0	0	-0.07198
ICAM3	0	0	0	0.12494
IFI16	0	0	0	0.11534
IGSF10	0	0	0.180122	0
IGSF9	0	0.092881	0	0
IL15RA	0	0	0	0.098764
IL16	0	0	0	0.120179
IL18R1	0	0	0	0.151059
IL18RAP	0	0	0	0.144011
IL1R2	0	0	0	0.105857
IL2RB	0	0	0	0.122858
IL2RG	0	0	0	0.135668
IL33	0	0	0	0.097482
IL34	0	0	0	0.102645
INHBA	0	0	0.183166	0
IQCH	0	-0.12317	0	0
IRF4	0	0	0	0.125196
IRX5	0	0	0	-0.07518
ITGA1	0	0	0.220379	0
ITGBL1	0	0	0.202693	0
ITM2A	0	0	0	0.104359
ITPRIPL1	0	0	0	0.171457
JAM2	0	0	0.213782	0
JAM3	0	0	0.278763	0
KANK2	0	0	0.184282	0
KAT2A	0	0	-0.15583	0
KCNE4	0.052258	0	0	0
KCTD12	0	0	0.253038	0
KDF1	0	0	-0.20271	0

KIAA1109	0	-0.15858	0	0
KIF17	0.041179	0	0	0
KIF19	0	0	0	0.100498
KIF22	0	0	-0.14534	0
KIF3A	0	-0.12502	0	0
KIF3B	0	-0.11159	0	0
KIFC3	0	0.069343	0	0
KLF16	0	0.116148	0	0
KLHL12	0	0	0	-0.08712
KLHL17	0	0.094703	0	0
KLHL29	0	0	0	0.116653
KRTCAP2	0	0	-0.12996	0
KRTCAP3	0	0	-0.19737	0
L3MBTL4	0	0	0	0.096651
LAGE3	0	0.072932	0	0
LAMA2	0	0	0.279675	0
LAMA4	0	0	0.290977	0
LAMB1	0	0	0.243621	0
LAMP3	0	0	0	0.144271
LAPTM4B	-0.04336	0	0	0
LATS2	0	0	0.202929	0
LAYN	0	0	0.183921	0
LCAT	0	0.071528	0	0
LCK	0	0	0	0.153915
LDB2	0	0	0.215589	0
LEO1	0	0	0	-0.08506
LGALS2	0	0	0	0.16417
LGR6	0	0	0	0.101722
LIMD2	0	0.091362	0	0.111742
LIMK1	0	0.085443	0	0
LIX1L	0	0	0	0.107761
LMNB2	0	0.180161	0	0
LPAR2	0	0.113328	0	0
LRBA	0	-0.17962	0	0
LRCH1	0	0	0.192122	0
LRCH2	0	0	0.325584	0
LRP1	0	0	0.26927	0
LRRC15	0	0	0.194579	0
LRRC32	0	0	0.213497	0
LRRC45	0	0	-0.14313	0
LRWD1	0	0.101479	-0.13936	0
LSM4	0	0	-0.22901	0
LSR	0	0.076895	-0.13499	0
LTBP2	0	0	0.214388	0
LUM	0	0	0.273873	0
LYPLA2	0	0.070264	0	0

MAB21L1	0	0	0.184132	0
MAF	0	0	0.189495	0
MAGED2	0	0	0	-0.06602
MAGEF1	0	0	-0.12837	0
MAGIX	0	0	-0.14082	0
MAL	0	0	0	0.15289
MAP3K14	0	0	0	0.115412
MAP3K6	0	0.095604	0	0
MAPK7	0	0.08228	0	0
MARCKSL1	0	0.084511	0	0
MBTPS2	0	-0.23138	0	0
MCM10	-0.04967	0	0	0
MCM4	-0.04578	0	0	0
MCM5	0	0.075	0	0
MCTP2	0	0	0	0.113638
MDH2	0	0	-0.16539	0
MDM2	0	-0.15739	0	0
MEA1	0	0	-0.12034	0
MEF2B	0	0.086709	0	0
MEF2C	0	0	0.202233	0
METTL2A	0	0	0	-0.06711
MEX3A	0	0.10164	0	0
MEX3D	0	0.220882	0	0
MFAP2	0	0.067994	0	0
MFNG	0	0	0	0.100241
MFSD3	0	0	-0.12614	0
MGAT3	0	0	0	0.107656
MIB2	0	0.071585	0	0
MID1	0	0	0	0.107476
MITF	0	0	0.194579	0
MLF2	0	0	-0.21485	0
MLST8	0	0	-0.14751	0
MMP19	0	0	0.191311	0
MOB3B	0	0	0	0.113266
MON2	0	-0.1558	0	0
MORF4L2	0	0	0	-0.07046
MRPL12	0	0	-0.15622	0
MRPL14	0	0	-0.20836	0
MRPL17	0	0	-0.13353	0
MRPL35	0	0	0	-0.06348
MRPL36	0	0	-0.12249	0
MRPL37	0	0	-0.11974	0
MRPL55	0	0	-0.12259	0
MRPS33	0	0	-0.12874	0
MRPS34	0	0	-0.13095	0
MSH3	0	-0.13928	0	0

MSRB3	0	0	0.293665	0
MTA3	0	0	0	-0.0657
MXRA5	0	0	0.28845	0
NAT1	0.059052	0	0	0
NCS1	0	0.087279	0	0
NDUFA7	0	0	-0.13222	0
NDUFAB1	0	0	-0.13492	0
NDUFB11	0	0	-0.14147	0
NDUFS3	0	0	-0.12076	0
NDUFS8	0	0	-0.13234	0
NEBL	0	-0.14291	0	0
NECAB3	0	0	0	-0.06745
NEGR1	0	0	0.178581	0
NELL2	0.039612	0	0	0
NETO2	-0.04391	0	0	0
NEXN	0	0	0.186293	0
NGFR	0	0	0	0.097977
NHP2	0	0	-0.12214	0
NID1	0	0	0.271813	0
NLRP1	0	0	0	0.122034
NMB	0	0.069541	0	0
NPM3	0	0.100733	0	0
NR2C2AP	0	0.07049	-0.15733	0
NR2F6	0	0	-0.12918	0
NRG2	0	0	0	0.111135
NRP1	0	0	0.232997	0
NUAK1	0	0	0.214971	0
NUCB2	0	-0.15285	0	0
NUDT1	0	0.096584	0	0
NUP62	0	0.070676	0	0
OCIAD2	0	0	-0.12552	0
OGN	0	0	0.192191	0
OLFML1	0.073275	0	0.346366	0
OLFML2B	0	0	0.199432	0
OMD	0	0	0.246454	0
OSR1	0	0	0	0.096817
OXER1	0	0.069305	0	0.101927
P4HA3	0	0	0.180046	0
PACS2	0	0.078381	0	0
PAK3	0	0	0	0.105924
PANK3	0	-0.13824	0	0
PAPLN	0	0	0	0.125035
PBX4	0	0.089408	0	0
PCDH18	0	0	0.262952	0
PCDHGA9	0	0	0.179342	0
PCDHGB7	0	0	0.197641	0

PCSK5	0	0	0.272158	0
PDCD2L	0	0	-0.12206	0
PDE12	0	-0.13408	0	0
PDE1A	0	0	0.193577	0
PDE3A	0	0	0.208542	0
PDGFRA	0	0	0.272297	0
PDGFRB	0	0	0.290038	0
PDIK1L	0	-0.1054	0	0
PDLIM7	0	0.08718	0	0
PEAK1	0	0	0.256736	0
PFDN2	0	0.090723	-0.13803	0
PFDN6	0	0	-0.16617	0
PFN1	0	0.083963	0	0
PGAM5	0	0	-0.17973	0
PGGT1B	0	-0.19273	0	0
PGK1	-0.05001	0	0	0
PIAS4	0	0.098567	0	0
PIGK	0	-0.11953	0	0
PIGM	0	0	0	-0.09243
PIK3CB	0	-0.10594	0	0
PIM1	0	0	0	0.099272
PIP4K2C	0	0	0	-0.07656
PKD2	0	0	0.22067	0
PKMYT1	0	0.074934	0	0
PKP3	0	0	-0.19171	0
PLA2G12A	0	-0.14332	0	-0.06573
PLA2G2D	0	0	0	0.112238
PLCG2	0	0	0	0.14932
PLD4	0.054889	0	0	0
PLSCR4	0	0	0.205917	0
PLXNC1	0	0	0.28011	0
PNO1	-0.06509	0	0	0
PNPO	0	-0.13431	0	0
PODN	0	0	0.234963	0
POLD1	0	0.126255	0	0
POLR2H	0	0	-0.15256	0
POLR2J	0	0	-0.126	0
POMC	0	0.068205	0	0
POP1	-0.04333	0	0	0
POP7	0	0	-0.22484	0
POSTN	0	0	0.257838	0
PPIH	0	0.077048	0	0
PPM1G	0	0	-0.12858	0
PPP1R12C	0	0.08235	0	0
PPP1R14B	0	0.147827	0	0
PPP1R15A	0	0.082064	0	0

PPP1R16B	0	0	0	0.123358
PPP2R2B	0	0	0	0.143793
PRDM8	0	0	0.17818	0.124479
PRDX2	0	0	-0.12749	0
PREX2	0	0	0.215081	0
PRKD1	0	0	0.228991	0
PRR15	0	0	0	-0.08494
PRR19	0	0	-0.14019	0
PRR5	0	0.086814	0	0
PRR7	0	0.069203	-0.12148	0
PRRC1	0	-0.11329	0	0
PRSS53	0	0.06843	0	0
PSMB4	0	0	-0.12074	0
PSMG3	0	0.0853	0	0
PSRC1	0	0	-0.14514	0
PTGDS	0	0	0	0.133226
PTGER4	0	0	0	0.104445
PTK7	0	0.104441	0	0
PTPN7	0	0	0	0.133735
PTPRB	0	0	0.190682	0
PTPRG	0	0	0.207521	0
PTPRT	0.040839	0	0	0
PTX3	0	0	0	0.104882
PYCR2	0	0	-0.12434	0
R3HDM4	0	0.096303	0	0
RAB3A	0	0	0	-0.06734
RAB7B	0	0	0	0.102348
RAC2	0	0	0	0.106158
RAI2	0.05202	0	0	0
RANGAP1	0	0	-0.13464	0
RARRES1	0	0	0	0.122769
RASEF	0	-0.19225	0	0
RASGRF2	0	0	0.317645	0
RASGRP2	0	0	0	0.136687
RBBP8NL	0	0	-0.15707	0
RBL2	0	-0.13454	0	0
RBM18	0	-0.12667	0	0
RBMS3	0	0	0.310911	0
RCAN2	0	0	0.205926	0
RCSD1	0	0	0	0.130334
RECK	0	0	0.341625	0
RECQL4	0	0	-0.14011	0
REXO1	0	0.171854	0	0
RFC2	0	0	-0.12212	0
RFTN2	0	0	0.298508	0
RGMA	0	0.078505	0	0.098046

RHOJ	0	0	0.180177	0
RICTOR	0	-0.11644	0	0
RITA1	0	0	-0.13294	0
RNASEH2A	0	0	-0.16109	0
RNF103	0	-0.1332	0	-0.10506
RNF166	0	0.090111	0	0
RPL13	0	0.083491	0	0
RPP40	-0.04893	0	0	0
RPS6KA3	0	0	0	0.10314
RPUSD1	0	0	-0.17019	0
RRM2B	0	-0.23309	0	0
RTKN	0	0.096083	-0.17305	0
RTN1	0.060493	0	0	0
RTN4R	0	0.073072	0	0
RUNDC1	0	-0.11426	0	-0.0708
RUNX1T1	0	0	0.322776	0
RUNX2	0	0	0.209118	0
RUVBL1	0	0	-0.14127	0
RUVBL2	0	0	-0.14533	0
S100A11	0	0.069428	0	0
S100B	0	0	0	0.111285
SAC3D1	0	0.075411	-0.16497	0
SAMD1	0	0.105978	0	0
SAMD8	0	-0.17615	0	0
SAR1B	0	-0.21126	0	0
SCAMP1	0	-0.19566	0	0
SCARA5	0	0	0	0.096516
SCNM1	0	0.07868	0	0
SEC16A	0	0	0	-0.07154
SEC62	0	-0.14824	0	0
SELL	0	0	0	0.124006
SELP	0	0	0	0.101199
SEMA3D	0	0	0.22272	0
SEMA3G	0.040689	0	0	0
SEMA5A	0	0	0.22503	0
SEPHS1	-0.05798	0.074953	0	0
SERPINF1	0	0	0.179787	0
SERPINH1	0	0.095367	0	0
SFRP1	0	0	0	0.113445
SFRP2	0	0	0.208755	0
SH3BP1	0	0.07173	0	0
SH3D19	0	0	0.180244	0
SHOX2	0	0	0.19614	0
SIRPG	0	0	0	0.137502
SLC19A1	0	0	-0.11954	0
SLC22A3	0	0	0	0.098943

SLC25A10	0	0	-0.17287	0
SLC25A22	0	0	-0.15432	0
SLC25A39	0	0	-0.24852	0
SLC29A4	0	0.078356	0	0
SLC2A10	0	0	0	-0.07937
SLC30A5	0	-0.14295	0	0
SLC30A9	0	-0.11847	0	0
SLC35F5	0	-0.11454	0	0
SLC39A3	0	0.078707	0	0
SLC39A6	0	-0.16314	0	0
SLC39A9	0	-0.11257	0	-0.0684
SLC9A3R1	0	0	0	-0.06458
SLC9A9	0	0	0	0.100476
SLIT2	0	0	0.288862	0
SLIT3	0	0	0.245346	0
SMARCA4	0	0.115197	0	0
SMTN	0	0.097508	0	0
SNAI2	0	0	0.195956	0
SNRNP70	0	0.071061	0	0
SNRPB	0	0	-0.13725	0
SNRPC	0	0	-0.1353	0
SNRPD1	0	0	-0.12381	0
SNX20	0	0	0	0.119864
SORD	0	0	0	-0.08775
SPARC	0	0	0.238729	0
SPARCL1	0	0	0.221536	0
SPDEF	0	0	0	-0.07446
SPN	0	0	0	0.118519
SPON1	0	0	0.261583	0
SPPL2B	0	0.095771	0	0
SPR	0	0	0	-0.07637
SPTY2D1	0	-0.13664	0	0
SRGN	0	0	0	0.097769
SRP54	0	-0.10942	0	-0.08789
ST6GAL2	0	0	0.21772	0
ST8SIA1	0	0	0	0.12417
STAC	0	0	0	0.105326
STAP2	0	0.081743	0	0
STARD10	0	0	0	-0.06972
STARD8	0	0	0.21263	0
STARD9	0	0	0.195797	0
STAT4	0	0	0	0.167899
SUCO	0	0	0	-0.06587
SUSD3	0.039958	0	0	0
SVEP1	0	0	0.256097	0
SYAP1	0	0	0	-0.0737

SYCE1L	0	0.094083	0	0
SYNE1	0	0	0.229127	0
SYT8	0	0.07213	0	0
SYT9	0.043885	0	0	0
SYTL2	0	-0.11989	0	0
SYTL4	0.045127	0	0	0
TACC1	0	0	0.180032	0
TACR1	0	0	0	0.116189
TAF1C	0	0.083769	0	0
TAF9B	0	-0.12494	0	0
TBC1D9	0	-0.15628	0	0
TBX15	0	0	0.277996	0
TBX18	0	0	0.281997	0
TBX5	0	0	0.252544	0
TCEAL7	0	0	0.19036	0
TCF4	0.073155	0	0	0
TCF7L1	0	0.084244	0	0
TCP11L2	0	-0.12758	0	0
TEK	0	0	0.18036	0
TFF1	0.040463	0	0	0
TGFBR2	0	0	0.278473	0
TGFBR3	0.046332	0	0	0
THBS2	0	0	0.233395	0
THOC6	0	0.077575	0	0
THSD4	0	-0.15409	0	0
TIGIT	0	0	0	0.125982
TIMM10	0	0	-0.13165	0
TIMM13	0	0.094792	0	0
TIMM17B	0	0	-0.19474	0
TIMP2	0	0	0.2383	0
TK1	0	0	-0.13486	0
TLE4	0	0	0	0.149285
TLL1	0	0	0.257338	0
TLR4	0	0	0.197577	0
TMBIM6	0	-0.15545	0	-0.11498
TMCO1	0	0	0	-0.08146
TMED2	0	0	0	-0.06702
TMED7	0	-0.30282	0	0
TMEM106B	0	-0.16055	0	0
TMEM132A	0	0.085126	0	0
TMEM147	0	0	-0.13178	0
TMEM177	0	0	-0.12025	0
TMEM220	0	0	0	0.103896
TMEM240	0	0.082368	0	0
TMEM259	0	0.08509	0	0
TMEM71	0	0	0	0.149237

TMEM87B	0	-0.15994	0	0
TMEM9B	0	-0.10641	0	0
TMSB10	0	0.139848	0	0
TMTC3	0	-0.11009	0	0
TNFAIP3	0	0	0	0.135318
TNFRSF12A	0	0.069989	0	0
TNFRSF1B	0	0	0	0.135971
TNFRSF25	0	0.084664	0	0
TNFRSF8	0	0	0	0.144391
TNPO1	0	-0.11405	0	0
TNS1	0	0	0.220945	0
TOMM40	0	0.079418	-0.16297	0
TONSL	0	0.075333	0	0
TOX2	0	0	0	0.100304
TPD52	0	0	0	-0.06328
TPI1	0	0	-0.12655	0
TRABD2A	0	0	0	0.171647
TRAF3IP3	0	0	0	0.139927
TRIM22	0	0	0	0.097404
TRIP10	0	0.147399	0	0
TRMT10A	0	0	0	-0.06574
TSEN54	0	0	-0.18255	0
TSFM	0	0	-0.13661	0
TSHZ2	0	0	0.205594	0
TSHZ3	0	0	0.316755	0
TSPAN13	0	0	0	-0.1155
TTYH3	0	0.117801	0	0
TUBA3D	0.055193	0	0	0
TUBG1	0	0	-0.1792	0
TUFM	0	0	-0.15224	0
TWF1	0	-0.10384	0	0
TXNL4A	0	0	-0.12379	0
TYK2	0	0.101025	0	0
U2AF1L4	0	0.07759	0	0
UACA	0	0	0.181605	0
UBE2M	0	0	-0.16169	0
UBE2S	0	0	-0.17859	0
UBE2W	0	-0.10828	0	0
UBE3A	0	-0.1534	0	0
UCK2	-0.06278	0	0	0
UCKL1	0	0	-0.11987	0
UEVLD	0	-0.17067	0	0
UHMK1	0	-0.13416	0	0
UQCC2	0	0	-0.14898	0
UQCC3	0	0	-0.12986	0
USP38	0	-0.1331	0	0

USP44	0	0	0	0.111838
VCAN	0	0	0.313879	0
VGLL3	0	0	0.235032	0
VPS13C	0	-0.14145	0	0
VPS72	0	0.082382	-0.12651	0
VSTM4	0	0	0.294481	0
WDFY4	0	0	0	0.110178
WDR86	0	0	0	0.096864
WNT6	0	0.109111	0	0
WRNIP1	0	0	-0.17278	0
WWP1	0	-0.20653	0	0
XIAP	0	-0.16631	0	0
YBX3	0	0.071285	0	0
YDJC	0	0.068094	-0.15626	0
YIPF6	0	-0.27385	0	-0.10097
YWHAQ	-0.04714	0	0	0
ZBTB17	0	0.094687	0	0
ZBTB42	0	0	0	-0.07669
ZCCHC24	0	0	0.229144	0
ZEB1	0.074771	0	0.406956	0
ZEB2	0	0	0.301237	0
ZFHX4	0	0	0.28479	0
ZFPM2	0	0	0.326115	0
ZMYND19	0	0.074165	-0.16699	0
ZNF148	0	-0.12295	0	0
ZNF24	0	-0.13291	0	0
ZNF316	0	0.075069	0	0
ZNF385B	0.043343	0	0	0
ZNF423	0	0	0.190666	0
