# Supplementary material for: Biomarker potential of the LEF1/TCF family members in breast cancer: Bioinformatic investigation on expression and clinical significance
Source: Genet Mol Biol. 2023 Dec 15;46(4):e20220346. doi: 10.1590/1678-4685-GMB-2022-0346 (PMC10723634; doi:10.1590/1678-4685-GMB-2022-0346)
Supplement: Table S3 - [file 1415-4757-GMB-46-4-e20220346-s3.pdf]

Genetics and Molecular Biology
https://doi.org/10.1590/1678-4685-GMB-2022-0346

Supplementary Material to "Biomarker potential of the LEF1/TCF family members in breast cancer: Bioinformatic investigation on expression and clinical significance"

Table S3 – Enrichment analysis of each regulon. Top 25 REACTOME pathways enriched to (A) *LEF1* regulon, (B) *TCF3* regulon, (C) *TCF4* regulon, and (D) *TCF7* regulon genes.

Supplementary table 3A

Reactome pathway	FDR values	Genes in overlap
Cell Cycle	1.47E-3	*CCNE1, CENPN, CENPO, YWHAQ, TUBA3D, MCM4, MCM10, DKC1, DSCC1*
Signaling by Rho GTPases, Miro GTPases and *RHOBTB3*	1.47E-3	*CCNE1, CENPN, CENPO, YWHAQ, TUBA3D, CCT6A, EVL, FGD3, ARHGDIB*
Cell Cycle Checkpoints	3.64E-3	*CCNE1, CENPN, CENPO, YWHAQ, MCM4, MCM10*
Formation of tubulin folding intermediates by CCT/TriC	3.77E-3	*TUBA3D, CCT6A, CCT5*
Protein folding	4.48E-3	*CCNE1, TUBA3D, CCT6A, CCT5*
rRNA processing	4.48E-3	*DKC1, DCAF13, PNO1, RPP40 BYSL*
Cooperation of Prefoldin and TriC/CCT in actin and tubulin folding	4.48E-3	*CCNE1, CCT6A, CCT5*
Association of TriC/CCT with target proteins during biosynthesis	6.53E-3	*CCNE1, CCT6A, CCT5*
Chromosome Maintenance	1.16E-2	*CENPN, CENPO, DKC1, DSCC1*
RHO GTPases Activate Formins	1.16E-2	*CENPN, CENPO, TUBA3D, EVL*
Folding of actin by CCT/TriC	1.59E-2	*CCT6A, CCT5*
rRNA modification in the nucleus and cytosol	1.59E-2	*DKC1, DCAF13, PNO1*
RHO GTPase Effectors	1.92E-2	*CENPN, CENPO, YWHAQ, TUBA3D, EVL*
Cell Cycle Mitotic	2.85E-2	*CCNE1, CENPN, CENPO, TUBA3D, MCM4, MCM10*

Supplementary table 3B

Reactome pathway	FDR values	Genes in overlap
Diseases of signal transduction by growth factor receptors and second messengers	6.07E-4	*CLTC, FRS2, CSK, CDC37, ERLEC1, PIK3CB, RICTOR, PRR5, ESR1, MDM2, CEBPB, MIB2, CSNK1A1, AGGF1*
Signaling by Rho GTPases, Miro GTPases and RHOBTB3	5.98E-3	*CLTC, FRS2, CSK, CDC37, PFN1, CFL1, LIMK1, CDC20, CENPT, TRIP10, TWF1, ARHGEF1, RTKN, FAF2, SH3BP1, BAIAP2L*
Transport of small molecules	1.37E-2	*CLTC, ERLEC1, SAR1B, MBTPS2, LSR, LCAT, WWP1, ATP6V1A, SLC39A6, SLC39A3, SLC30A5, CLCN3, TTYH3, ATP8B1, SLC29A4*
PI3K/AKT Signaling in Cancer	1.37E-2	*FRS2, PIK3CB, RICTOR, PRR5, ESR1, MDM2*
Nervous system development	1.37E-2	*CLTC, FRS2, PIK3CB, PFN1, CFL1, LIMK1, MAPK7, SMARCA4,*

		PDLIM7, RPL13, EFNA4, LYPLA2, RGMA
Plasma lipoprotein assembly, remodeling, and clearance	1.37E-2	*CLTC, SAR1B, MBTPS2, LSR, LCAT*
Signaling by Receptor Tyrosine Kinases	1.37E-2	*CLTC, FRS2, CSK, CDC37, PIK3CB, RICTOR, PRR5, ESR1, WWP1, ATP6V1A, MAPK7, BAX*
Cell Cycle	1.37E-2	*MDM2, CDC20, CENPT, RBL2, E2F4, BTRC, PIAS4, NUP62, CDT1, POLD1, MCM5, TNPO1, PKMYT1, CENPW*
RNA Polymerase II Transcription	1.37E-2	*RICTOR, PRR5, ESR1, MDM2, CEBPB, WWP1, SMARCA4, BAX, RBL2, E2F4, TAF9B, CTR9, POMC, RRM2B, DDIT4, TCF7L1, ALYREF, U2AF1L4, THOC6, NR2C2AP, EAF1*
RHO GTPase cycle	1.37E-2	*CLTC, FRS2, CSK, CDC37, TRIP10, TWF1, ARHGEF1, RTKN, FAF2, SH3BP1, BAIAP2L2*
RET signaling	1.37E-2	*FRS2, PIK3CB, MAPK7, PDLIM7*
Zinc transporters	1.63E-2	*SLC39A6, SLC39A3, SLC30A5*
Adaptive Immune System	1.63E-2	*CLTC, CSK, PIK3CB, RICTOR, PRR5, MIB2, CDC20, SAR1B, WWP1, BTRC, UBE2W, FBXL19, KIF3A, KIF3B, UBE3A*
Post-translational protein modification	1.63E-2	*ESR1, MDM2, CDC20, SAR1B, BTRC, PIAS4, NUP62, TAF9B, CTR9, UBE2W, FBXL19, ARF3, ARF5, TMED7, B4GALT2, RNF103, EDEM3, DNMT3A, THSD4, TMEM132A, PIGK*
TP53 Regulates Transcription of Genes Involved in G2 Cell Cycle Arrest	1.63E-2	*BAX, RBL2, E2F4*
Metabolism of lipids	1.63E-2	*PIK3CB, SAR1B, MBTPS2, PIAS4, POMC, ARF3, CHD9, SAMD8, CERS6, ACER2, GPD2, PLA2G12A, CPNE3, HACD3*
Unfolded Protein Response (UPR)	2.17E-2	*CEBPB, MBTPS2, CREBRF, CXXC1, ZBTB17*
Cellular responses to stimuli	2.46E-2	*MDM2, CEBPB, MBTPS2, ATP6V1A, MAPK7, RPL13, NUP62, CHD9, CREBRF, CXXC1, ZBTB17, PPP1R15A, SERPINH1, STAP2*
COPI-dependent Golgi-to-ER retrograde traffic	2.85E-2	*KIF3A, KIF3B, ARF3, ARF5, TMED7*
Transcriptional Regulation by TP53	2.99E-2	*RICTOR, PRR5, MDM2, BAX, RBL2, E2F4, TAF9B, RRM2B, DDIT4*
Constitutive Signaling by AKT1 E17K in Cancer	3.56E-2	*RICTOR, PRR5, MDM2*
Metal ion SLC transporters	3.56E-2	*SLC39A6, SLC39A3, SLC30A5*
Cell Cycle Mitotic	3.96E-2	*CDC20, CENPT, RBL2, E2F4, BTRC, NUP62, CDT1, POLD1, MCM5, TNPO1, PKMYT1*
G1/S-Specific Transcription	4.53E-2	*RBL2, E2F4, CDT1*

Supplementary table 3C

Reactome pathway	FDR values	Genes in overlap
Extracellular matrix organization	3.52E-21	*COL3A1, COL5A1, COL5A2, COL1A1, COL1A2, LAMB1, HSPG2, DCN, LAMA2, LAMA4,*

		SPARC, LUM, VCAN, ASPN, COL8A1, COL12A1, COL15A1, COL14A1, TLL1, MMP19, ADAMTS5, NID1, TIMP2, ADAM12, P4HA3, DDR2, ITGA1, JAM2, JAM3, LTBP2
ECM proteoglycans	4.63E-13	*COL3A1, COL5A1, COL5A2, COL1A1, COL1A2, LAMB1, HSPG2, DCN, LAMA2, LAMA4, SPARC, LUM, VCAN, ASPN*
Degradation of the extracellular matrix	4.63E-13	*COL3A1, COL5A1, COL5A2, COL1A1, COL1A2, LAMB1, HSPG2, DCN, COL8A1, COL12A1, COL15A1, COL14A1, TLL1, MMP19, ADAMTS5, NID1, TIMP2*
Signaling by Receptor Tyrosine Kinases	2.2E-11	*COL3A1, COL5A1, COL5A2, COL1A1, COL1A2, LAMB1, LAMA2, LAMA4, SPARC, ADAM12, HGF, THBS2, CDK5, NRP1, CXCL12, POLR2H, POLR2J, MEF2C, PDGFRA, PDGFRB, MLST8, FGF7, FLRT2, ATP6V0B, AXL, PCSK5*
MET activates PTK2 signaling	3.12E-10	*COL3A1, COL5A1, COL5A2, COL1A1, COL1A2, LAMB1, LAMA2, LAMA4, HGF*
Collagen biosynthesis and modifying enzymes	1,00E-09	*COL3A1, COL5A1, COL5A2, COL1A1, COL1A2, COL8A1, COL12A1, COL15A1, COL14A1, TLL1, P4HA3*
MET promotes cell motility	5.07E-9	*COL3A1, COL5A1, COL5A2, COL1A1, COL1A2, LAMB1, LAMA2, LAMA4, HGF*
Non-integrin membrane-ECM interactions	5.24E-9	*COL3A1, COL5A1, COL5A2, COL1A1, COL1A2, LAMB1, HSPG2, LAMA2, LAMA4, DDR2*
Assembly of collagen fibrils and other multimeric structures	6.6E-9	*COL3A1, COL5A1, COL5A2, COL1A1, COL1A2, COL8A1, COL12A1, COL15A1, COL14A1, TLL1*
Collagen chain trimerization	7.03E-9	*COL3A1, COL5A1, COL5A2, COL1A1, COL1A2, COL8A1, COL12A1, COL15A1, COL14A1*
Integrin cell surface interactions	8.06E-9	*COL3A1, COL5A1, COL5A2, COL1A1, COL1A2, HSPG2, LUM, COL8A1, ITGA1, JAM2, JAM3*
Collagen degradation	8.15E-9	*COL3A1, COL5A1, COL5A2, COL1A1, COL1A2, COL8A1, COL12A1, COL15A1, COL14A1, MMP19*
Collagen formation	1.29E-8	*COL3A1, COL5A1, COL5A2, COL1A1, COL1A2, COL8A1, COL12A1, COL15A1, COL14A1, TLL1, P4HA3*
Mitochondrial translation	4.19E-7	*TSFM, MRPL55, MRPL37, MRPS33, MRPL12, MRPL17, MRPL14, MRPL36, MRPS34, TUFM*
Signaling by MET	1.08E-6	*COL3A1, COL5A1, COL5A2, COL1A1, COL1A2, LAMB1, LAMA2, LAMA4, HGF*
Diseases of glycosylation	1.52E-6	*HSPG2, DCN, LUM, VCAN, ADAMTS5, THBS2, SEMA5A, OMD, OGN, SPON1, ALG3*
RHO GTPase cycle	8.31E-6	*PREX2, RASGRF2, ARHGAP31, ARHGAP20, ARHGAP24, STARD8,*

		RTKN, ARHGAP6, NDUFS3, DOCK11, FERMT2, CCT7, PEAK1, UACA, HSPE1, RHOJ, AKAP12
Laminin interactions	8.55E-6	*LAMB1, HSPG2, LAMA2, LAMA4, NID1, ITGA1*
Developmental Biology	2.22E-5	*COL3A1, COL5A1, COL5A2, LAMB1, LAMA2, ITGA1, CDK5, NRP1, CXCL12, POLR2H, POLR2J, MEF2C, SEMA5A, ARPC1A, PSMB4, PLXNC1, ANK2, CNTN1, SLIT2, SLIT3, FOXO1, E2F1, KAT2A, ZFPM2, FLI1, PKP3, EBF1*
Diseases associated with glycosaminoglycan metabolism	5.43E-5	*HSPG2, DCN, LUM, VCAN, OMD, OGN*
Signaling by Rho GTPases, Miro GTPases and RHOBTB3	6.61E-5	*PREX2, RASGRF2, ARHGAP31, ARHGAP20, ARHGAP24, STARD8, RTKN, ARHGAP6, NDUFS3, DOCK11, FERMT2, CCT7, PEAK1, UACA, HSPE1, RHOJ, AKAP12, ARPC1A, CENPM, RANGAP1*
Syndecan interactions	1.29E-4	*COL3A1, COL5A1, COL5A2, COL1A1, COL1A2*
Nervous system development	2.03E-4	*COL3A1, COL5A1, COL5A2, LAMB1, LAMA2, ITGA1, CDK5, NRP1, CXCL12, SEMA5A, ARPC1A, PSMB4, PLXNC1, ANK2, CNTN1, SLIT2, SLIT3*
Diseases of metabolism	2.33E-4	*HSPG2, DCN, LUM, VCAN, ADAMTS5, THBS2, SEMA5A, OMD, OGN, SPON1, ALG3*
Signaling by PDGF	3.51E-4	*COL3A1, COL5A1, COL5A2, THBS2, PDGFRA, PDGFRB*

Supplementary table 3D

Reactome pathway	FDR values	Genes in overlap
Cytokine Signaling in Immune system	2.01E-13	*LCK, FYN, CXCL1, TNFRSF1B, RPS6KA3, S100B, IL33, CXCL2, GATA3, IL2RG, IL2RB, IL15RA, STAT4, FCER2, IL1R2, PIM1, IRF4, PTPN7, IL18R1, IL18RAP, IL34, IL16, HSPA9, MAP3K14, CD40, CD27, TNFRSF8, TRIM22, MID1*
Signaling by Interleukins	2.14E-12	*LCK, FYN, CXCL1, TNFRSF1B, RPS6KA3, S100B, IL33, CXCL2, GATA3, IL2RG, IL2RB, IL15RA, STAT4, FCER2, IL1R2, PIM1, IRF4, PTPN7, IL18R1, IL18RAP, IL34, IL16, HSPA9*
Innate Immune System	2.28E-8	*LCK, FYN, CXCL1, TNFRSF1B, RPS6KA3, S100B, MAP3K14, RAC2, PLCG, SELL, RASGRP2, FGR, CD3G, PAK3, RAB3A, C3, CFP, ACLY, FCN1, CHI3L1, CANT1, PTX3, ICAM3, TNFAIP3, CLEC10A, C1R, NLRP1, IFI16*
Negative regulation of the PI3K/AKT network	6.63E-6	*LCK, FYN, IL33, RAC2, EGFR, NRG2, ERBB3, ESR1, PIP4K2C*
Chemokine receptors bind chemokines	1.19E-5	*CXCL1, CXCL2, CXCR6, CCR4, CXCR3, CCL21, CX3CL1*

Hemostasis	1.73E-5	*LCK, FYN, GATA3, RAC2, PLCG2, SELL, RASGRP2, FGR, KIF19, SELP, SIRPG, SPN, GYP, CD2, CD48, CYB5R1, MAGED2, SRGN*
Generation of second messenger molecules	1.84E-5	*LCK, PLCG2, CD3G, PAK3, CD3D, CD3E*
Costimulation by the CD28 family	4.11E-5	*LCK, FYN, MAP3K14, CD3G, PAK3, CD3D, CD3E*
Constitutive Signaling by Aberrant PI3K in Cancer	5.26E-5	*LCK, FYN, RAC2, EGFR, NRG2, ERBB3, ESR1*
Membrane Trafficking	1,00E-04	*CD3G, RAB3A, EGFR, KIF19, CD3D, TACR1, AP1M2, ARF3, TMED2, COPZ1, SEC16A, RAB7B, ARL1, GOLGA5, YIPF6, TPD52*
Vesicle-mediated transport	1.21E-4	*CD3G, RAB3A, EGFR, KIF19, CD3D, TACR1, AP1M2, ARF3, TMED2, COPZ1, SEC16A, RAB7B, ARL1, GOLGA5, YIPF6, TPD52, SCARA5*
Cell surface interactions at the vascular wall	2.22E-4	*LCK, FYN, SELL, SELP, SIRPG, SPN, GYPC, CD2, CD48*
Peptide ligand-binding receptors	2.43E-4	*CXCL1, CXCL2, C3, CXCR6, CCR4, CXCR3, CCL21, CX3CL1, TACR1*
PI3K/AKT Signaling in Cancer	2.57E-4	*LCK, FYN, RAC2, EGFR, NRG2, ERBB3, ESR1*
Class A/1 (Rhodopsin-like receptors)	3.26E-4	*CXCL1, CXCL2, C3, CXCR6, CCR4, CXCR3, CCL21, CX3CL1, TACR1, OXER1, PTGER4*
Interleukin-2 family signaling	4.79E-4	*LCK, IL2RG, IL2RB, IL15RA, STAT4*
Interleukin-10 signaling	5.64E-4	*CXCL1, TNFRSF1B, CXCL2, FCER2, IL1R2*
Intracellular signaling by second messengers	9.04E-4	*LCK, FYN, IL33, RAC2, EGFR, NRG2, ERBB3, ESR1, PIP4K2C, MTA3*
Signaling by GPCR	9.04E-4	*CXCL1, RPS6KA3, CXCL2, RASGRP2, C3, EGFR, CXCR6, CCR4, CXCR3, CCL21, CX3CL1, TACR1, OXER1, PTGER4, FGD2*
PD-1 signaling	1.28E-3	*LCK, CD3G, CD3D, CD3E*
Platelet activation, signaling and aggregation	1.28E-3	*LCK, FYN, RAC2, PLCG2, RASGRP2, SELP, CYB5R1, MAGED2, SRGN*
Neutrophil degranulation	1.28E-3	*CXCL1, TNFRSF1B, SELL, FGR, RAB3A, C3, CFP, ACLY, FCN1, CHI3L1, CANT1, PTX3*
Signaling by ERBB4	1.28E-3	*S100B, EGFR, NRG2, ERBB3, ESR1*
Adaptive Immune System	1.28E-3	*LCK, FYN, MAP3K14, CD40, PLCG2, SELL, RASGRP2, CD3G, PAK3, C3, ICAM3, CD3D, CD3E, AP1M2, CD79A, CD79B*
CD28 co-stimulation	2.19E-3	*LCK, FYN, MAP3K14, PAK3*
